# The influence of trematode parasite burden on gene expression in a mammalian host

**DOI:** 10.1186/s12864-016-2950-5

**Published:** 2016-08-11

**Authors:** Bhagya K. Wijayawardena, Dennis J. Minchella, J. Andrew DeWoody

**Affiliations:** 1 Department of Biological Sciences, Purdue University, West Lafayette, Indiana 47907 USA; 2 Departments of Biological Sciences, Forestry and Natural Resources, Purdue University, West Lafayette, Indiana 47907 USA

**Keywords:** Differential expression, Xenobiotics, RNAseq, Illumina HiSeq, Immune response, *Schistosoma mansoni*, Non-target sequences

## Abstract

**Background:**

Parasites can profoundly impact their hosts and are responsible for a plethora of debilitating diseases. To identify global changes in host gene expression related to parasite infection, we sequenced, assembled, and annotated the liver transcriptomes of Balb/cj mice infected with the trematode parasite *Schistosoma mansoni* and compared the results to uninfected mice. We used two different methodologies (i.e. *de novo* and reference guided) to evaluate the influence of parasite sequences on host transcriptome assembly.

**Results:**

Our results demonstrate that the choice of assembly methodology significantly impacted the proportion of parasitic reads detected from the host library, yet the presence of non-target (xenobiotic) sequences did not create significant structural errors in the assembly. After removing parasite sequences from the mouse transcriptomes, we analyzed host gene expression under different parasite infection levels and observed significant differences in the associated immunologic and metabolic responses based on infection level. In particular, genes associated with T–helper type 1 (Th–1) and T–helper type 2 (Th–2) were up-regulated in infected mice whereas genes related to amino acid and carbohydrate metabolism were down-regulated in infected mice. These changes in gene expression scale with infection status and likely impact the evolutionary fitness of hosts.

**Conclusions:**

Overall, our data indicate that a) infected mice reduce the expression of key metabolic genes in direct proportion to their infection level; b) infected mice similarly increase the expression of key immune genes in response to infection; c) patterns of gene expression correspond to the pathological symptoms of schistosomiasis; and d) identifying and filtering out non-target sequences (xenobiotics) improves differential expression prediction. Our findings identify parasite targets for RNAi or other therapies and provide a better understanding of the pathology and host immune repertoire involved in response to *S. mansoni* infections.

**Electronic supplementary material:**

The online version of this article (doi:10.1186/s12864-016-2950-5) contains supplementary material, which is available to authorized users.

## Background

Flatworm parasites of the genus *Schistosoma* are the causative agents of schistosomiasis in humans and other mammals. Schistosomiasis is a widespread tropical disease that affects over 200 million people in the tropics, causing severe morbidity and mortality in infected individuals [1, 2]. *S. mansoni* is the most widespread schistosome, its distribution ranging from the old world to the new world [3, 4]. *S. mansoni* is a blood parasite that navigates through the viscera via the host circulatory system. The *S. mansoni* life cycle involves a mammalian definitive host, where sexual reproduction occurs, and a snail intermediate host that provides a vehicle for asexual propagation [5]. The free–swimming microscopic larvae (cercariae) are released from freshwater snails and infect the mammalian host by penetrating unbroken skin. After several days, the parasites exit the cutaneous tissue through blood vessels and travel first to the lungs and then into the systemic vasculature and finally to the hepatic portal system [6, 7]. Parasites mature, mate, and lay eggs within 5–6 weeks of definitive host infection. Parasite eggs that pass through the mammalian intestinal wall are excreted with host feces and subsequently infect the freshwater snail of the genus *Biomphalaria*, thus completing the life cycle.

Parasite eggs carried by the vascular system of the mammalian host can become lodged in host tissues, mainly liver. The parasite egg antigens can induce a severe host immune response resulting in multi-cellular granulomatous inflammations surrounding the eggs [5]. This inflammatory reaction destroys parasite eggs and sequesters secreted toxins, but also causes host liver damage. The pathology of schistosomiasis is a result of severe fibrosis, caused by granuloma formation [8]. The granulomatous response occurs through several pathological stages that ultimately lead to vascular obstructions which increase portal blood pressure and foster the development of portal–systemic venous shunts [9]. The development of these granulomas is attributed to various cytokines and chemokines associated with the T–helper type 2 (Th–2) immune response [10]. Ultimately, the parasite eggs are calcified and the granuloma develops into a fibrous plaque [11].

Schistosomiasis pathology depends on the parasite burden, ranging from a few scattered granuloma to systemic portal fibrosis [12, 13]. No study (to our knowledge) has comprehensively evaluated the immunogenetic response of a mammalian host in response to schistosome burden, yet such research could help combat schistosomiasis. Our primary objective was to compare global host gene expression in uninfected and in *S. mansoni*–infected mice to study parasite–induced changes in gene expression. We discovered significant differences in gene expression among differentially infected hosts, and these changes corresponded to the pathological response of the hosts to the parasites.

Our secondary objective was to critically evaluate the effect of non-target (xenobiotic) sequences on host transcriptome assembly. Eukaryotic organisms are effectively diverse ecosystems within themselves, as multiple species are typically found in such close association that physical separation may be impossible. For example, extracts from a given human tissue could also contain DNA from viral, bacterial, fungal, or invertebrate (parasite) sources. Thus, it is no great surprise that sequenced libraries often contain DNA from non-target sources such as parasites, pathogens, commensals and prey items [14]. The overall influence of these contaminants on host gene reconstruction is not fully appreciated [15], but non-target sequences can adversely affect downstream analysis by leading to inaccurate assemblies that ultimately produce false gene predictions [16]. We tested the extent to which non-target (e.g., *S. mansoni*) reads compromise estimates of host gene expression at each stage of the RNAseq analysis procedure by sequencing, assembling, and annotating transcriptomes of experimental (infected) and control (uninfected) mice. We used both reference–based (i.e. mapping of reads against the genomic reference) and *de novo* transcriptome assembly procedures and found that the latter retrieved significantly more non-target sequences. The results from the non-target analysis should be of interest to the broader genomics community, as they are relevant to the study of virtually all multicellular eukaryotes.

## Methods

### Experimental design, library construction, and sequencing

**Fig. 1:**
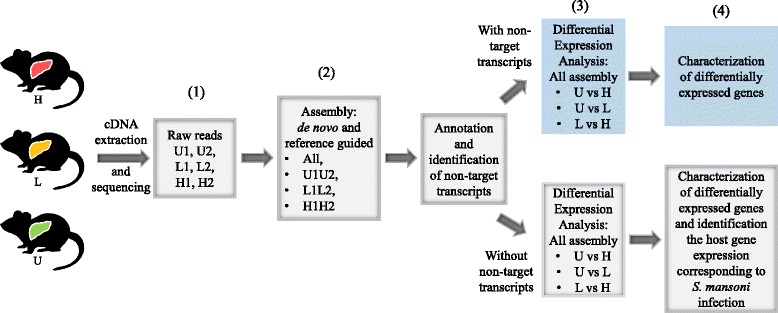
The workflow. We followed the primary steps in NGS transcriptome analysis (1: Generating raw reads, 2: Assembling reads, 3: Differential expression analysis and 4: characterization of differentially expressed genes) to understand the influence of non-target reads on transcriptome analysis. First, Liver cDNA of mice infected with the parasite *S. mansoni* (High dose infection: H, low dose infection: L and uninfected: U; 2 mice per group) were sequenced with Illumine HiSeq. Then, sixteen transcriptomes were assembled using both *de novo* (Trinity *de novo* and SOAPdenovo–Trans) and reference based (Genome guided Trinity and Cufflinks) approaches at the different infection levels, to identify the influence of parasite reads (i.e. xenobiotics) on transcriptome assembly. Subsequently, the transcriptomes were annotated and putative non-target transcripts were identified. Then differential expression was calculated, before and after filtering the transcripts of non-target origin, between infected and uninfected mice and between mice infected at different parasite loads. Finally after removing non-target reads from the transcriptomes the host gene expression corresponding to *S. mansoni* infection was characterized

Our workflow is summarized in Fig. 1. Briefly, seven-week old full–sib, BALB/cJ male mice were obtained from the Jackson Laboratories (Bar Harbor, Maine). We cultured *S. mansoni* of the NMRI strain (originally from Puerto Rico) in *Biomphalaria glabrata* snails [17]. Infected snails were exposed to fluorescent light for ~2 hrs to induce cercarial emergence [17]. Mice were then infected with 50 cercariae (*n* = 6 mice designated L1–L6; lower parasite burden); and 200 cercariae (*n* = 6 mice designated H–H6; higher parasite burden). Six uninfected mice (U1 –U6) were used as controls. Mouse infections were conducted by immersing their tails in water containing cercariae for ~2 hrs [18]. Cercarial numbers and viability were determined using light microscopy prior to infection. Cercariae water was examined post–exposure to make sure that approximately the desired number of cercariae passed into the mouse host. Seven weeks post–infection, all mice were euthanized. The parasite maintenance and mouse infections were carried out following a protocol approved by Purdue Animal Care and User Committee (protocol 1111000225).

Immediately after euthanization, mice were dissected and the right lobe of the liver was used for standard RNA extractions with Trizol reagent (Invitrogen). Dissections and extractions were conducted using sterile techniques in a laminar flow hood to help avoid potential human contamination. RNA quality and quantity were assessed via gel electrophoresis, spectrophotometry (Nanodrop 8000; Thermo Scientific), and Agilent Bioanalyzer 2100. cDNA library preparation and barcoding followed the Illumina TruSeq RNA sample preparation kit protocol. Thereafter, a subset of 6 samples (U1, U2, L1, L2, H1 and H2) was sequenced using an Illumina HiSeq2000 (Illumina 1 lane; six paired–end libraries; read length 100 bp) at the Purdue genomics core facility.

After sequencing, we conducted a number of quality control measures. Adaptors and low quality reads were clipped using Trimmomatic [19]; poor quality bases (< Phred–20) were removed from both the 5′ and 3′ ends of the reads. All reads <30 nt in length were discarded. The FastQC v0.11.2 [20] software v11.2 was used to assess and visualize the quality of the remaining reads. To help identify the parasite reads from among all the raw reads, BLASTN (v2.2.3, %ID >90 %; E–value = 10^-12^) searches were carried out against the *S. mansoni* transcriptome ([21, 22]; BLASTN %ID >90 %; E–value = 10^-12^).

### Transcriptome assembly, annotation, and characterization

**Fig. 2:**
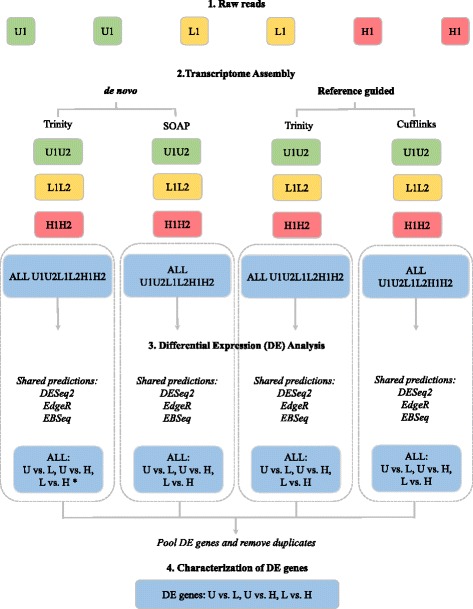
The pipeline. Sixteen transcriptomes were assembled using both *de novo* (Trinity *de novo* and SOAPdenovo–Trans) and reference based (Genome guided Trinity and Cufflinks) approaches, to reflect different infection levels (U1U2, L1L2, H1H2 and Master assembly: All U1U2L1L2H1H2). Only the four master assemblies were used in Differential Expression (DE) analysis, comparing the three treatments (U vs. L, U vs. H and L vs. H). Shared DE predictions of three DESeq2, EdgeR and EBSeq were identified for each transcriptome assembly. Before characterizing the DE genes using Gene ontology analysis, DE genes were pooled across assemblies and duplicates were removed. *: Trinity *de novo* master assembly was used for hierarchical clustering and qPCR validation

Different computational approaches produce transcriptomes that vary dramatically in quality, and there is no *a priori* “best” assembler for a given data set [23]. Thus, we employed four algorithms representing both *de novo* (Trinity *de novo* r20140717 [24] and SOAPdenovo–Trans 1.03 [25]) and reference guided (Genome guided Trinity r20140717 [24] and Cufflinks v2.2.1 [26]) transcriptome assembly approaches (Additional file 1: Table S1). We assembled trimmed reads into transcriptomes as detailed in Fig. 2. To improve transcriptome recovery, multiple k–mer SOAPdenovo–Trans libraries (k–mers 21, 25, 29, 33, 37) were constructed and combined using the Cd–hit program [25, 27]. Genome–guided Trinity (with GSNAP aligner) and Cufflinks (with TopHat 2.0.13 aligner; [28]) were used to construct guided assemblies referenced to publicly available mouse genome data (Ensembl: GCA_000001635.5_GRCm38.p3_genomic.fna). To assemble and identify *S. mansoni* reads, a separate set of transcriptomes was assembled with Genome–guided Trinity using the *S. mansoni* genome as a reference.

The presence of xenobiotics may impact transcriptome assembly statistics and accuracy by (inadvertently) combining reads from multiple species. To help identify such errors, the four relevant data partitions (i.e., U1U2, L1L2, H1H2, and ALL; Fig. 2) were evaluated using four assemblers and QUAST v.2.3 was used to calculate descriptive statistics of each [29]. A metric of assembly contiguity, N50, indicates the length of which half of all nucleotides in the assembly are comprised of sequences of equal or longer length. However, N50 values can be misleading because transcript lengths are highly heterogeneous. Therefore, we compared our assembly N50 values to the N50 value of the *Mus musculus* mRNA data set to obtain a N50 ratio (N50 assembly/N50 mouse mRNA). Thus, a N50 ratio of 1 would indicate perfect recovery of mouse transcripts [30]. To evaluate the assembly completeness, each assembly was compared against a set of core eukaryotic genes (CEGs) using CEGMA v2.5 [31].

The Ortholog Hit Ratio (OHR) is an estimate of the amount of a transcript contained in each unigene (i.e. contig and scaffold), and it quantifies the completeness of each transcript [32]. For each unigene, the OHR was calculated by dividing the length of the putative coding region of each unigene by the total length of the ortholog for that unigene. Orthologs were identified by comparing each unigene against the uniprot mouse and *S. mansoni* proteins (BLASTX; E–value: 10^–6^; Alignment length ≥90 %). The resulting best BLASTx hits were considered orthologs and the hit region in the contig was considered to be a putative coding region [32]. In addition, scripts available through the MUMMer package [33] were used to find the presence of structural variants such as inversions (part of a contig reversed with respect to the reference gene), translocations, relocations (within a gene), duplications, and insertions and deletions (indels) compared to the Ensemble *Mus musculus* cDNA data set [30, 34].

All transcriptomes were annotated using BLASTX [21] via the BLASTER tool [35] against the Swissprot database with the E–value < 10^–6^ and percentage identity ≥90 % as the quality thresholds. Newly characterized transcriptomes may encode proteins that are lacking detectable homologies to known proteins. To capture those coding regions, TransdDecoder (http://transdecoder.github.io/) was used to identify candidate protein–coding regions based on nucleotide composition, open reading frame (ORF) length, and Pfam domain content.

To assess parasite contamination in the host assemblies, the top BLASTX hits (*n* = 1000; E–value 10^–6^; [21]) were collected for each transcript and the proportion of contigs yielding only non–vertebrate hits were calculated. Furthermore, each empirical transcriptome and ORF set (TransdDecoder; http://transdecoder.github.io/) was compared against host and parasite reference transcriptomes to determine the origin of each contig (Ensemble: Mus_musculus.GRCm38.cdna.all.fa, UCSC refMrna.fa; [21, 22]). To validate the inferred presence of xenobiotics (i.e., non-target organisms identified by our bioinformatic analyses), we used PCR in an attempt to amplify expressed non-target reads from mouse liver cDNA samples (U1, U2, L1, L2, H1, H2; primer sequences: Additional file 1: Table S1). We used universal vertebrate [36] and *Schistosoma* species–specific primers [37] as positive controls to confirm the presence of high quality cDNA.

### Differential Expression (DE)

DE analysis was conducted using only the master assemblies (i.e. ALL reads) produced by each assembler as illustrated in Fig. 2. Briefly, trimmed Illumina reads for each individual library were mapped back to the appropriate master transcriptome assembly using Bowtie within the program RSEM [38, 39] to estimate the number of reads mapped to each contig. Only transcripts with at least 10 cumulative mapping counts were used in this analysis. DESeq2 [40], EdgeR [41], and EBSeq [42] were all used to evaluate differential expression (DE). All three software packages use a negative binomial distribution to account for overdispersion in transcriptome data sets. DESeq2 is conservative and uses a heuristic approach to detect outliers while avoiding false positives [40]. EdgeR follows a similar hypothesis testing approach, but uses a linear gene–wise dispersion estimation method that results in higher sensitivity at the cost of increased false positives [41, 43]. EBSeq uses a Baysian framework to determine DE and exhibits moderate sensitivity [42, 43]. Each DE analysis was composed of three pairwise comparisons; U vs. L, U vs. H, and L vs. H. Differentially expressed genes were identified after a correction for false discovery rate (FDR 0.05; [44]). By following a conservative approach (e.g., of only selecting the genes predicted as differentially expressed by all three DE analysis software packages), we sought to minimize false positives and only identify host genes that were truly differentially expressed in response to parasite infection (see “shared predictions” in Fig. 2).

### Characterization of differentially expressed genes

Each differentially expressed transcript was compared to the *S. mansoni* genome and transcriptome (BLASTN, E = 10^–6^; Percentage Identity ≥90 %; [21]) to identify the origin of differentially expressed transcripts. The transcripts that match with *S. mansoni* were compared against mouse genome and transcriptome databases (Ensemble: Mus_musculus.GRCm38.cdna.all.fa, UCSC refMrna.fa) to differentiate possible xenobiotics from transcripts that are similar (i.e., conserved) between species. Transcripts with no significant matches to mouse were considered to be of parasite/non-target origin. These transcripts were then further gauged by conducting BLASTN and BLASTX searches against Swissprot, Genbank EST, nucleotide, and reference sequences. The DE analyses were repeated after all putative non-target transcripts (i.e. the transcripts that matched the *S. mansoni* transcriptome, but not the mouse transcriptome) were filtered out from the assemblies and reads were remapped back to the transcriptomes. The DE predictions were then compared before and after removing xenobiotic–like sequences to test the bioinformatics consequences of inadvertently including parasite sequences in host transcriptomes.

Annotated genes (BLASTX, E–value: 10^-6^; Percentage ID: ≥90 %) that were identified as differentially expressed by all three DE software packages were selected separately for each assembly. After removing duplicates between assemblies, these genes were collectively used for gene set enrichment analysis. The DAVID Bioinformatics Resource [45] was used to assign GO terms and KEGG pathway IDs [46] to all annotated contigs. Subsequently, significantly overrepresented GO terms were identified using Fisher’s exact test and after correcting for false discovery rate (FDR; [44]).

### Gene expression in uninfected vs. infected mice

Transcripts of mouse origin (i.e. after removing possible *S. mansoni*–like reads) were used to identify host genes responding to *S. mansoni* infections. REVIGO [47] was used to summarize and visualize the lists of significantly enriched GO terms. REVIGO condenses the GO terms by finding a representative subset of the GO terms using a clustering algorithm that removes functional redundancies. This results in a smaller number of representative terms for ease of handling and interpretation. To further characterize the mouse liver transcriptome, we searched for the genes corresponding to immune response, against differentially expressed genes and compared their expression level between the treatments (U vs. L, U vs. H, and L vs. H). To identify common patterns of gene expression between all treatments, hierarchical clustering was conducted using the Pearson correlation coefficient (after normalizing transcript counts using a variance stabilizing transformation) on 861 Trinity *de novo* transcripts over and under expressed ≥ log_2_ 2. Thereafter, the identified clusters were annotated using GO terms [45]. All statistical analyses were implemented in the statistical analysis programming language R (www.r-project.org). Unless otherwise specified, default parameters of the software/modules were used.

To validate a subset of the genes we inferred to be DE, we performed real–time quantitative PCR (cDNA samples; 6 biological replicates per U, L, and H treatment). Primers corresponding to 10 DE genes (4 up-regulated and 6 down-regulated) were designed using Primer 3 software (http://bioinfo.ut.ee/primer3-0.4.0/; Additional file 1: Table S2). Hypoxanthine phosphoribosyltranferase (HPRT) was used as a housekeeping gene for relative quantification [48]. Additionally, primers corresponding to *Mus musculus* cytokines IL–1, IL–6, IL–13, IL–5, IL–4 and IL–10 were sourced from the literature and used in qPCR analysis to gauge cytokine response to infection [49, 50]. The thermal profile for all genes was 95 °C for 10 min, followed by 40 cycles of 95 °C for 15 s and 59 °C for 1 min. A melting curve analysis was conducted from 50 °C to 90 °C with 0.5 °C increases per cycle for a total of 80 cycles to insure there was no mis–annealing or contaminated cDNA in the sample. Each reaction was performed in three replicates and alongside a no–template negative control to rule out contamination. For each sample, threshold cycle numbers required to reach a predetermined fluorescence value were measured and compared with that of the control gene, in an effort to correct for PCR efficiencies. Subsequently, the expression ratios were tested for significance using the freely available REST software and 10,000 randomizations [51]. Comparisons between the treatments (U vs. L, U vs. H, and L vs. H) and methodology (qPCR and RNAseq gene expression ratios of Trinity *de novo* DESeq2) were carried out using Pearson’s correlation coefficient.

## Results

### Transcriptome assembly, annotation and characterization

**Fig. 3:**
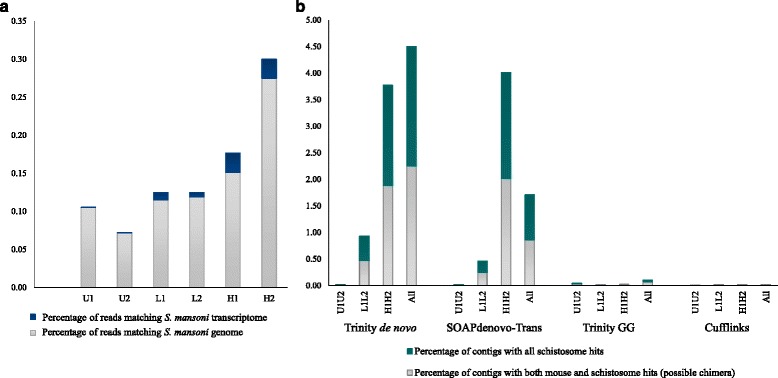
Percentage of parasite reads in mouse raw read libraries and assemblies **a** Percentage of raw reads matching *S. mansoni*. Concordant matches are represented (BLASTN %ID >90 %; E–value = 10^–12^) **b** Possible non-target reads in assemblies. Assemblies were compared against mouse and *S. mansoni* transcriptomes to identify non-targets (BLASTN E–value: 10^–6^; Percentage identity ≥90 %). de novo assemblies (Trinity de novo, SOAPdenovo–Trans) contained more non-target reads compared to reference guided assemblies (Reference guided trinity, Cufflinks)

Our sequencing effort produced ~60 million reads from each of six mouse livers representing three biological treatments (see Additional file 1: Tables S3–S4 for descriptive statistics). After discarding 1–2 % of the reads from each library due to poor quality sequence (Additional file 1: Table S4), we aimed to differentiate host reads from parasite reads (see Additional file 1: Table S4; Fig. 3(a)). Across all libraries, fewer than 0.3 % the overall reads matched (BLASTN %ID >90 %; E–value = 10^-12^) *S. mansoni* and, conversely, more than 99.7 % matched the mouse (Fig. 3a). The *de novo* assemblies contained significantly more parasite transcripts than the reference-based assemblies, and the percentage of non-host reads increased with increased infection intensity (Fig. 3b).

To evaluate assembly quality we assessed CEG complements, structural variants, the OHRs, and used BLAST searches. All assemblies contained >89 % of the known CEG genes (See Additional file 1, Results: Assembly evaluation for further details), indicating that our sequencing depth was sufficient for transcriptome reconstruction. Neither assembly algorithm (*de novo* vs. reference guided) nor infection level seemed to overly influence the presence of structural variants. We observed no difference in the OHRs between *de novo* and reference guided assemblies. However, the transcriptome assemblies of highly infected mice (H1H2) generally contained unigenes with low OHRs, suggesting the presence of incomplete transcripts. As expected, the taxonomic distribution of BLASTX hits indicated more non–chordate hits for the H1H2 assembly than the U1U2 assembly (especially in *de novo* assemblies). These findings suggest that *de novo* approaches may more effectively identify non-target transcripts, but the *de novo* assemblies also included more contigs that matched both mouse and *S. mansoni* transcripts (i.e., potential synthetic chimeras; Fig. 3(b)).

In an attempt to reconstruct portions of the *S. mansoni* transcriptome using the non-target hits, we performed a guided assembly using the *S. mansoni* genome as a reference. The overall number of transcripts, the number of transcripts matching *S. mansoni* mRNA, and the number of transcripts with non–chordate BLASTX hits increased with increasing parasite burden (Additional file 1: Tables S11–S12). Most of the non–chordate hits were to *S. mansoni* proteins curated in Swissprot. Collectively, these data indicate that we captured parasite transcripts directly from host tissue.

### Differentially expressed (DE) genes

Differentially expressed transcripts identified in both *de novo* and reference–guided assemblies showed significant matches to both the *S. mansoni* genome and transcriptome (see Additional file 1: Figure S1). All *S. mansoni*–like DE transcripts in reference–guided libraries also yielded significant matches to the mouse genome and transcriptome, suggesting that these sequences represent highly conserved regions. Some *S. mansoni*–like DE transcripts in *de novo* assemblies did not show significant matches to the mouse (Table 1; 171 unique transcripts in total, 93 Trinity *de novo* and 78 SOAPdenovo–Trans transcripts). All of the *S. mansoni*-like transcripts were identified in infected mice but never identified in uninfected mice, suggesting the transcripts were from the parasite or from associated non-target organisms. The percentage of these xenobiotic contigs in DE transcripts varied from 0–2.5 % (Table 1). The highest number of non-targets was identified by edgeR. Approximately 15 % of the non-targets contigs were differentially expressed as gauged by all three DE programs.

**Table 1:** Number and percentage (xenobiotic reads/DE transcripts) of non-target reads predicted to be differentially expressed (DE). Non-targets were identified in the differentially expressed transcripts assembled using *de novo* methods (Trinity *de novo* and SOAPdenovo–Trans). These were represented only in the cDNA libraries of infected or highly infected hosts

Software	Number of *S. mansoni* sequences in DE genes (Percentage)					
	UH Down	UH Up	UL Down	UL Up	LH Down	LH Up
DESeq2						
Trinity *de nov*o	0	28 (1 %)	0	0	0	0
SOAPdenovo–Trans	0	43 (1.2 %)	0	5 (0.3 %)	1 (0.1 %)	3 (0.6 %)
edgeR						
Trinity *de nov*o	0	92 (2.5 %)	0	8 (0.5 %)	0	2 (0.7 %)
SOAPdenovo–Trans	0	77 (1.8 %)	0	14 (0.6 %)	0	1 (0.2 %)
EBSeq						
Trinity *de nov*o	0	7 (0.3 %)	0	0	0	3 (0.3 %)
SOAPdenovo–Trans	0	12 (0.3 %)	0	4 (0.2 %)	1 (0.06 %)	9 (0.9 %)

UH: U vs H; UL: U vs L; LH: L vs H

U: Uninfected; L: Low–infected; H: High–infected

Up: Up regulated; Down: Down regulated

To further characterize these DE non-targets transcripts, we analyzed their alignment lengths (i.e., the length of the match with a corresponding *S. mansoni* transcript), their shared identity with *S. mansoni* transcripts, and their expression level (log_2_ fold change). On average, ~500 bases of non-target reads matched *S. mansoni* transcripts and identity exceeded 90 % (Additional file 1: Figures S2–S3). The inferred expression level of non-target transcripts varied, but many of them were highly expressed (mean fold change of 6.6; Additional file 1: Figure S4). When we compared the GC content of the parasite and host transcriptomes, we found 36 and 50 % for *S. mansoni* and mouse, respectively (Additional file 1: Figure S6). The mean GC content of our putative xenobiotic transcripts was 41 % and hence more similar to *S. mansoni*. Based on these collective attributes, most of these reads are probably not derived from mouse cells but more likely represent true biological contaminants associated with *S. mansoni*. These sequences could be derived from the parasite or its symbionts, so we refer to them as non-target (i.e. xenobiotic) transcripts because they do not appear to be of mouse origin.

Approximately 81 % of the differentially expressed non-target transcripts had significant BLASTX matches (E–value: 10^–6^; 100 hits per query sequence; Additional file 1: Figure S5). BLAST searches against Genbank and Swissprot yielded *S. mansoni* sequences as the best hit for over 50 % of the non-target transcripts (Genbank nr and est: 99 %, refseq: 70 %, Swissprot: 55 %). Most of these xenobiotic transcripts represented *S. mansoni* 40S ribosomal proteins and egg antigen sequences.

To confirm that xenobiotic contigs were present due to the actual presence of parasite cDNA in our libraries (as opposed to bioinformatics assembly errors), we conducted PCR on mouse liver cDNA samples using universal primers. Amplification efficiency (as gauged by band intensity) increased with increasing infection level of the hosts, suggesting the presence of more non-target transcripts in highly infected hosts (Additional file 1: Figure S7). No xenobiotic amplification was observed in uninfected mouse samples.

We conducted DE analysis and characterization of differentially expressed reads before and after filtering out xenobiotic transcripts (i.e., 1208 non-target transcripts from Trinity *de novo*, 868 from SOAPdenovo–Trans and 35 from Genome–guided Trinity) from the assemblies (Figs. 1 and 2). This analysis revealed that non-target transcripts influence global DE predictions, mostly by increasing the number of DE transcripts (Additional file 1: Figure S8). Global GO and KEGG IDs were not significantly affected by the presence of xenobiotic–like sequences in the assemblies (Additional file 1: Table S13).

### Host immune response to *S. mansoni* infection

**Fig. 4:**
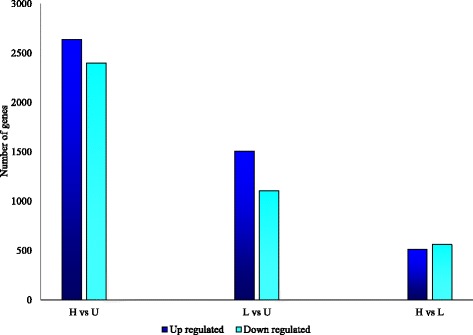
The number of DE genes used in characterization of mouse immune response against *S. mansoni* infection (High dose infection: H, low dose infection: L and uninfected: U; 2 mice per group). Four separate master transcriptomes were assembled using *de novo* (Trinity *de novo* and SOAPdenovo–Trans) and reference based (Genome guided Trinity and Cufflinks) approaches. Thereafter the non-target transcripts were identified and removed. Differential expression was calculated using DESeq2, EdgeR, and EBSeq and for each assembly shared predictions were extracted. The figure represents the resulting differentially expressed genes corresponding to the four assembly approaches, after removing the duplicates between assemblies

For our characterization of the host response to infection, we relied on the DE results from the mouse assemblies minus the non-target transcripts (see Fig. 4). The sample–to–sample distance calculated from the log–transformed gene–count matrix showed replicates clustering together, indicating low within–treatment variability in gene expression (Additional file 1: Figure S9; corresponds to Trinity *de novo*). Furthermore, the qPCR expression ratios were consistent with the RNAseq data (correlation coefficient =0.9; Additional file 1: Figure S10; corresponds to Trinity *de novo*). Together, these data provide confidence that our inferences regarding DE of host genes are robust.

**Fig. 5:**
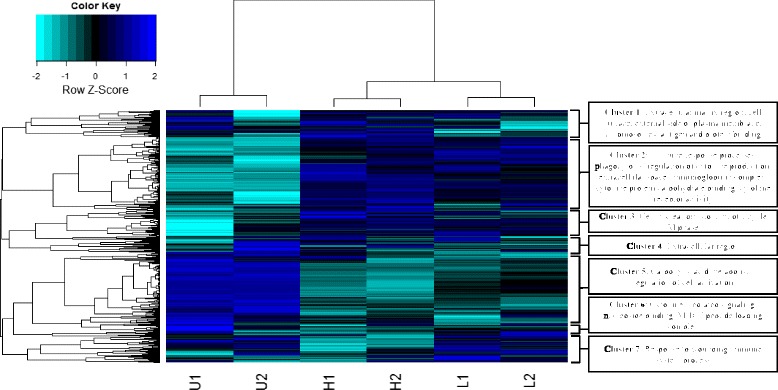
Gene expression patterns in *S. mansoni* infected mice. Hierarchical clustering identified two major expression patterns (up-regulation and down-regulation in infected mice compared to control uninfected mice), containing 9 different clusters. Gene expression patterns are observed between high infected (H1, H2) and low infected (L1, L2) mice. Gene expression patterns are represented as a heat map with relatively unchanged genes in black, down regulated genes in light blue and up regulated genes in dark blue. Clusters were annotated using DAVID and REVIGO functional annotation tools [45, 47]. U1U2: uninfected controls, L1L2: infected, low parasite burden, H1H2: infected, high parasite burden. Trinity *de novo* assembly was used

**Fig. 6:**
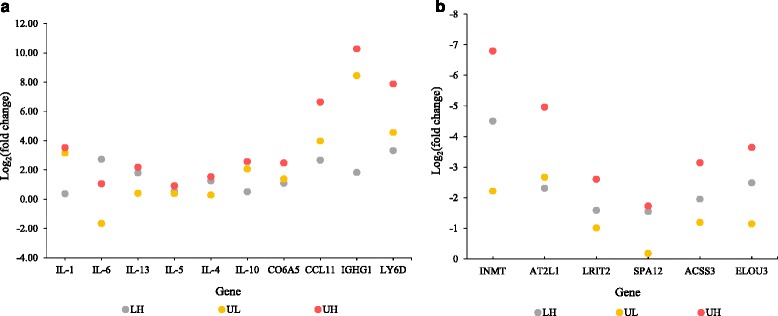
qPCR expression profiles of cytokines (IL–1, IL–6, IL–13, IL–5, IL–4, IL–10) and DE genes. Log _2_ of expression ratios were calculated compared to control (LH: H compared to U; UL: L compared to U; UH: H compared to U; [51]). **a** Cytokines and up–regulated genes. **b** Down–regulated genes. U: Uninfected; L: Low–infected; H: High–infected

These expression data show that the greatest number of DE transcripts were identified between U and H mice but significant differences in DE were also identified between L and H mice, illustrating that the infection level influences host gene expression (Figs. 5 and 6). GO enrichment analysis revealed that the GO terms corresponding to the “biological process category (e.g. “immune system process)” are highly represented in host DE gene sets (Additional file 1: Table S14).

We used REVIGO to summarize and visualize the significant expressed GO terms (Additional file 1: Figure S11–S15). Gene products of functional categories such as immune response, regulation of cytokine production, endocytosis, and immune system processes were significantly enriched due to the parasite infection (Additional file 1: Figure S11–S12; Additional file 1: Tables S15–S16). We identified significant differences in gene expression between mice that were infected with high and low parasite loads (Additional file 1: Figure S13; Additional file 1: Table S17). Specifically, the GO categories of cellular matrix organization, regulation of cell adhesion, and organ development were significantly up-regulated in H compared to L mice. In all three comparisons (U vs. H; U vs. L; and L vs. H) metabolism–related GO terms were significantly under–expressed (Additional file 1: Figure S14–S16; Additional file 1: Tables S18–S19). KEGG pathway analysis showed similar patterns; infected mice had increased expression of immune–related pathway IDs and decreased expression of metabolic pathway related KEGG IDs (Additional file 1: Tables S20–S25).

Hierarchical clustering identified the gene sets differentially expressed in infected mice compared to the uninfected. We identified seven different clusters that corresponded to different functional annotations (Fig. 5). Overall, genes corresponding to many metabolic pathways were down-regulated in infected mice whereas immune–related genes were up-regulated in infected mice. For example, genes associated with the extracellular matrix and immune system process were differentially expressed between L and H mice (Fig. 5). To identify the changes in gene expression corresponding to the infection status, we plotted the qPCR gene expression ratios of the three comparisons (U vs. H; U vs. L; and L vs. H; Fig. 6). The change in gene expression of many immune related genes (IL-1, IL-6, IL-13, IL-5, IL-4, IL-10, CO6A5, CCL11, IGHG1, LY6D, INMT, AT2L1, LRIT2, SPA12, ACSS3, ELOU3) corresponds to the parasite burden of the host, whereby highly infected hosts (i.e. H vs U) have a larger fold-change in gene expression than low-infected hosts (i.e. L vs U; Fig. 6). The same trend was observed when comparing the RNAseq gene expression ratios (Additional file 1: Table S26). Furthermore, the change in gene expression ratios corresponds to the parasite burden of the host (Additional file 1: Table S26, Fig. 6).

## Discussion

Our study documents key changes in host gene expression in response to parasite burden. Our data also indicate that transcripts from parasites and/or their symbionts can obfuscate host gene expression, and that due diligence is required to discriminate between host and xenobiotic, non-target transcripts. We first address the transcriptomic response of a mammalian host to trematode parasite burden, then briefly discuss issues associated with measuring gene expression in the context of xenobiotics.

### Host immune response to *S. mansoni* infection

After removing contigs of inferred non-target origin, we characterized the liver transcriptome of *S. mansoni* infected mice. Schistosomes are known to elicit both T–helper type 1 (Th–1) and T–helper type 2 (Th–2) immune responses [11, 52, 53]. During the first 4–6 weeks after infection, the Th–1 response is stimulated by the migration of immature adult worms [11] as characterized by increased levels of pro–inflammatory cytokines, including TNF–α, IL–1, IL–6 and IFN–γ. The inflammation caused by these cytokines can lead to the development of chronic infection and tissue scarring [54]. With the onset of egg-laying, Th–2 cytokines such as IL–4, IL–5, IL–10 and IL–13 begin to be expressed [11]. The Th–2 response peaks at ~8 weeks post–infection and subsequently decreases with progression of chronic infection (>12 weeks). We sacrificed mice 7 weeks after infection, and our expression profile includes both Th–1 and Th–2 cytokines (Additional file 1: Table S26; Fig. 6). One such cytokine, significantly up-regulated in our infected mice, is IL–10 (Additional file 1: Table S26; Fig. 6). IL–10 plays a key regulatory role in facilitating the shift from a Th–1 to Th–2 response and preventing the development of severe pathology due to excessive Th–1 response [53].

Much of the pathology of schistosomiasis is ascribed to the host granulomatous response induced by parasite eggs in the host liver [12]. Therefore, by sequencing the liver transcriptome we were able to characterize host genes expressed in response to parasite infection and the relative changes in their expression levels. Our results indicate that several GO categories (i.e., immune response, regulation of cytokine production endocytosis and immune system processes) are significantly enriched in infected hosts and these terms represent the diverse host responses to *S. mansoni* infection (Additional file 1: Figures S13–S16). Infected mouse livers show elevated expression of lymphocyte-associated proteins such as cell surface antigens (CDs; T cell, B cell, monocytes) and lymphocyte developmental factors (transforming growth factor, caspase recruitment domain family, SFFV proviral integration 1, IKAROS family zinc finger proteins) that mediate the innate immune response (Additional file 1: Tables S15–S17). The activation of B lymphocytes in infected mice may lead to the up-regulation of immunoglobulins (e.g., IgG and IgM), and the increased expression of toll–like receptors (TLRs) that we observed in infected mice may enable the development of hepatic fibrosis (Additional file 1: Tables S15–S17; [11, 55]. Beyond an innate response, the increased expression of major histocompatibility complex (MHC) class II genes that we observed in infected mice suggests the adaptive immune system stimulates antigen presentation to T–cells ([54]; Additional file 1: Tables S15–S17). Additional immunological enzymes (e.g. oligoadenylate synthetase, defensin), platelet receptors (p–selectin), and structural proteins of extracellular matrix (collagen, elastin, fibrillin) are also upregulated in infected hosts (Additional file 1: Tables S15–S17).

Schistosomiasis is known to cause significant damage to host liver function through hepatic fibrosis, in part by altering liver metabolism [56–59]. We observed that the enzymes involved in the production of acyl–CoA (acyl–CoA synthetase, acetyl–Coenzyme A acyltransferase), a key product of TCA cycle, were significantly under-expressed in infected mice (Additional file 1: Tables S18–S19). Our gene expression data from the mouse reinforce proteomic data and indicate a paucity of TCA cycle intermediates in infected hosts [59, 60]. Our gene expression data also suggest the parasite influences host amino acid metabolism by down–regulating enzymes involved in amino acid synthesis and catabolism (Additional file 1: Tables S18–S19). In addition, enzymes associated with the urea cycle (e.g., carbamoyl–phosphate synthetase), breakdown of toxin metabolites (e.g. UDP glycosyltransferase) and ion transport (3–hydroxybutyrate dehydrogenase) were also significantly downregulated in infected mice (Additional file 1: Tables S18–S19). Ultimately, such changes in gene regulation may impede host metabolism.

The severity of *S. mansoni* infection depends on parasite burden, the infection level affecting morphological behavioral, and physiological changes in the host [12, 13]. Mild infections often result in less severe clinical manifestations [12]. We infected mice with low (to mimic natural infections) and high parasite loads to identify differences in immune response associated with infection level. In terms of DE, transcriptomes of our H mice were significantly enriched with GO terms corresponding to extracellular matrix organization, regulation of cell adhesion, and organ development compared to L mice (Additional file 1: Figure S13). These processes represent tissue repair resulting from the Th–2 response. For instance, *S. mansoni* infected mice contain more collagen and enlarged livers (due to hepatosplenomegaly) than uninfected mice [8]. In addition, increasing parasite burden appears to increase metabolic costs as gauged by the relative expression of genes associated with metabolism (Fig. 5); such costs may impair host fitness and/or life span. Thus, our results confirm and quantify the relationship between parasite burden and host immunogenetic response [61, 62].

We observed the increased expression of type I and type II immune responses in highly infected hosts (Figs. 5 and 6; Additional file 1: Table S26). Specifically, components of the Th–2 response (IL–10, immunoglobulins) were significantly up-regulated in highly infected mice compared to low infected mice. Similar immunological changes have been observed in nematode–host systems, where an increased parasite burden results in the greater polarization of immune response from Th–1 to Th–2 [62, 63]. These response dynamics seem to be dosage dependent, triggered by parasite antigens. For instance, in the tapeworm *Echinococcus granulosus,* mild infections elicit both Th–1 and Th–2 responses whereas more intense infections result in Th–2 response. As Th–1 and Th–2 cells cross–regulate one another [54], the change in parasite dosage could alter both the nature and timing of the subsequent immune response. Our study was conducted at 7 week post-exposure when the parasite was beginning to lay eggs and the host was switching from a Th-1 to a Th-2 immune response. If this experiment was run earlier in the infection cycle, we predict that we would have only observed the up-regulation of genes associated with Th-1 response, whereas an experiment run later in the infection cycle would have yielded over-expression of genes associated with Th-2. Our results suggest that the immunological dynamics associated with schistosome infections may be characterized by a nonlinear dose–response function whereby low–dose infections elicit a much different host response than typical high–intensity laboratory infections.

### Characterization of parasite transcripts from host tissue

Our results indicate that non-target organisms can confound gene expression studies conducted at the transcriptome level, but heretofore the effects of such xenobiotics have not been evaluated [15, 16]. Presence of non-target transcripts (e.g., from the trematode parasite) constitute a very small fraction of the overall transcriptome, but can still significantly impact assembly, annotation, and gene expression assays.

Quality filtering is a crucial step in the analyses of massively parallel short–read sequence data, as filtering removes low quality reads, duplicate reads, and tag sequences to the benefit of the overall assembly [64]. Nevertheless, many non-target sequences pass such quality control measures (Additional file 1: Tables S3–S4). To identify *S. mansoni*–like sequences in our mouse liver datasets, we used both *de novo* and reference–based transcriptome assembly approaches. The *de novo* approach utilized sequencing redundancy to identify overlaps between the reads and subsequently assembles them into transcripts [23]. Despite its computational challenges, *de novo* assembly is usually most appropriate for non–model organisms because it does not rely on a reference genome. Nevertheless, *de novo* assembly is highly prone to template contaminations that occur in the presence of non-targets (Additional file 1: Tables S9–S10). Conversely, reference–based assemblies are sensitive, accurate (given the use of a high quality genome), and faster than *de novo* assembly [23]. Genome assemblies of model organisms such as the mouse are generally of high quality and therefore expected to facilitate a high quality transcriptome assembly. The presence of xenobiotics is less of a concern for reference–based libraries compared to *de novo* libraries, since non-target sequences are not expected to align with reference data and should end up on the “cutting room floor” as these assemblies discarded nearly a quarter of all high–quality reads [14]. Indeed, we identified DE non-target reads only in *de novo* assemblies (Table 1; Additional file 1: Tables S9–S10). Both Trinity *de novo* and SOAPdenovo–Trans captured non-target reads, possibly due to the high sensitivity of the multiple k–mer approach [24].

We tested for signatures of xenobiotic contamination in our RNAseq assemblies by estimating assembly correctness and evaluating completeness statistics. We found differences in GC content between putative mouse reads and putative non-target reads, and that the GC content in presumptive non-target reads was consistent with their derivation from *S. mansoni* (Additional file 1: Figure S6). Our results also indicate that datasets derived from a parasite–infected source tissue may contain less complete reads than more pure source tissue (Additional file 1: Table S9–S10). For instance, our H1H2 assemblies contained many more reads with an OHR ratio <0.8, indicating incomplete/truncated reads and/or novel transcripts (Additional file 1: Table S9). However, there were no apparent differences in the number of structural errors between assemblies (Additional file 1: Table S6).

Many putative *S. mansoni* reads that were DE were from long transcripts present in high copy numbers. Given that each mature female parasite produces ~300 eggs per day [5], we suggest these reads likely originated from *S. mansoni* eggs present in the livers of infected mice. This idea is buttressed by the fact that most of the putative non-target reads significantly matched to *S. mansoni* egg antigen sequences. In the presence of non-target sequences, DE analysis predicted more DE transcripts in infected hosts than in uninfected hosts (Additional file 1: Figure S8) but non-targets did not significantly change the outcome of GO and KEGG enrichment analysis.

Overall, the presence of non-target reads did not impact the host transcriptome assembly, but they did confound the analyses of DE (i.e. *de novo* assemblies, Table 1). We recommend removing all possible non-target reads from genome/transcriptome assemblies prior to DE analysis because an abundance of non-target sequences may negatively influence downstream analyses and lead to erroneous inferences [14]. We suggest filtering out such sequences using metagenomics databases, contaminant removal software (DeconSeq, QC-chain, [15, 16]), and genomic features such as GC content and codon usage bias to identify potential non-target sequences.

## Conclusions

Our data reveal general patterns of gene expression exhibited by mammalian hosts in response to parasite infection, and they highlight specific host biological processes most likely to be impacted by *S. mansoni*. After filtering parasites from the host transcriptomes, we identified and characterized genes that were differentially expressed among mice that varied in parasite burden. We determined that the up–regulation of genes related to the immune response and the down–regulation genes related to metabolism is proportional to the parasite burden of the host, whereby highly infected hosts exhibit more pronounced changes in gene expression relative to less infected hosts. These differences in gene expression reflect the pathological changes associated with *S. mansoni* infections and provide a better understanding of host–parasite interplay at the transcriptome level. Furthermore, our data highlight potential avenues for therapeutic intervention in the treatment of schistosomiasis (e.g., RNAi targets), and our experimental approach has broad utility for other host-parasite systems.

## Additional file

Additional file 1: Tables S1-S26 and Figures S1-S16. Contains additional results, primer sequences, RNA quality parameters, descriptive statistics and annotation details of assemblies, GO terms (lists and figures), significantly enriched KEGG IDs, xenobiotic characterizations, PCR results and qPCR validations. (PDF 2247 kb)
