# Supplementary material for: The influence of trematode parasite burden on gene expression in a mammalian host
Source: BMC Genomics. 2016 Aug 11;17:600. doi: 10.1186/s12864-016-2950-5 (PMC4982272; doi:10.1186/s12864-016-2950-5)
Supplement: Additional file 1: Tables S1-S26 and Figures S1-S16. — Contains additional results, primer sequences, RNA quality parameters, descriptive statistics and annotation details of assemblies, GO terms (lists and figures), significantly enriched KEGG IDs, xenobiotic characterizations, PCR results and qPCR validations. (PDF 2247 kb) [file 12864_2016_2950_MOESM1_ESM.pdf]

Additional file 1: Table of contents

Table/ Figure title		Page
Results	Assembly evaluation	1
Table S1	Transcriptome assembly	2
Table S2	Primer sequences developed for the analysis	3
Table S3	RNA quality	5
Table S4	Descriptive statistics of raw reads	6
Table S5	Descriptive statistics of transcriptome assemblies	7
Table S6	Structural errors in transcriptomes	10
Table S7	Average Ortholog hit ratio (OHR) of transcriptomes	11
Table S8	Assembly annotation statistics	12
Table S9	Comparing assemblies against mouse and *S. mansoni* transcriptomes	13
Table S10	Comparison of predicted ORFs against mouse and *S. mansoni* transcriptomes	14
Table S11	Annotation statistics of *S. mansoni*–guided Trinity assemblies	15
Table S12	BLASTN matches of *S. mansoni*–guided Trinity assemblies	15
Table S13	GO terms and KEGG IDs before and after filtering	16
Table S14	Representation of GO term categories in significantly expressed genes	16
Table S15	DE (upregulated in L compared to U) genes in GO term category immune system process.	17
Table S16	DE (upregulated in H compared to U) genes in GO term category immune system process.	21
Table S17	DE (upregulated H compared to L) genes in GO term category antigen binding	27
Table S18	DE (down–regulated in L compared to U) genes in GO term cytoplasmic part	27
Table S19	DE (down–regulated in H compared to U) genes in GO term category organic and metabolic processes	28
Table S20	KEGG pathway IDs over–expressed in high–infected livers compared to un–infected livers (U vs H).	30
Table S21	KEGG pathway IDs under–expressed in high–infected livers compared to un–infected livers (U vs H)	32
Table S22	KEGG pathway IDs over–expressed in low–infected livers compared to un–infected livers (U vs L).	34
Table S23	KEGG pathway IDs under–expressed in low–infected livers compared to un–infected livers (U vs L).	36

Table S24	KEGG pathway IDs over–expressed in high–infected livers compared to low–infected livers (L vs H).	38
Table S25	KEGG pathway IDs under–expressed in high–infected livers compared to low–infected livers (L vs H)	39
Table S26	Fold change (Log2 fold change) of immune response related genes	40
Figure S1	The number of DE genes predicted	41
Figure S2	Alignment length distribution of differentially expressed non-target reads	42
Figure S3	Percentage identity distribution of differentially expressed non-target reads	43
Figure S4	Fold change distribution of differentially expressed non-target reads	44
Figure S5	BLASTX matches of differentially expressed non-target reads	45
Figure S6	Percentage GC content of mouse and parasite reads	46
Figure S7	PCR amplification of non-target reads	48
Figure S8	The Change in number of DE transcripts after removing the reads of non-target origin.	49
Figure S9	Heat map representing sample to sample Euclidian distance	50
Figure S10	RNAseq vs qPCR gene expression correlation	51
Figure S11	GO "Biological processes" significantly over–expressed in low– infected livers compared to the uninfected livers	52
Figure S12	GO "Biological processes" significantly over–expressed in highly infected livers compared to the uninfected livers	53
Figure S13	GO "Biological processes" significantly over–expressed in high–infected livers compared to the low–infected livers	54
Figure S14	GO "Biological processes" significantly under–expressed in highly infected livers compared to the uninfected livers	55
Figure S15	GO "Biological processes" significantly under–expressed in low– infected livers compared to the uninfected livers	56
Figure S16	GO "Biological processes" significantly under–expressed in highly infected livers compared to the low–infected livers	57

Results: Assembly evaluation

The Trinity de novo, Trinity reference guided and SOAPdenovo-Trans assemblies were more complete than Cufflinks in terms of capturing CEGs (Core Eukaryotic Genes; Additional file 1: Table S5). However, all four approaches produced assemblies that contained >89% of CEG genes (Table S5). Other than SOAPdenovo–Trans H1H2 assembly containing many inversions compared to the other assemblies, there was no significant differences in structural variants between *de novo* and reference guided assemblies (Table S6).

The orthologue hit ratio (OHR) produces an estimate of transcript abundance represented by each unigene. We carried out BLASTX searches against both *Mus muculus* and *S. mansoni* protein databases to calculate the OHR. Most unigenes had high OHRs (≥0.8), indicating that many of the transcripts in our assemblies represent complete full length copies (Table S7). An OHR of >1, indicative of insertions in unigenes, was common in the Trinity genome–guided assemblies. The presence of incomplete transcripts reduces the OHR, and our H1H2 assemblies contain more unigenes with a low OHR, despite having a relatively high number of protein hits. We observed a similar trend when the OHR was calculated using *S. mansoni* proteins (Table S7).

We used the Swissprot database for transcript annotation as it is well curated with little redundancy and is highly integrated with other databases. Not surprisingly, the taxonomic distribution of BLASTX hits indicates more non–chordate hits for the H1H2 assembly than U1U2 assembly (Table S8). This trend was most notable in de novo assemblies, which produced twice as many exclusively non–chordate hits and more sequences matching only to *S. mansoni* (Table S9). A similar pattern was observed in ORFs, where de novo assemblies contained many reads that were exclusively from the parasite and the number of parasite reads increased with increasing parasite load (Table S10). These findings suggest that de novo approaches may more effectively identify non-target transcripts, but the de novo assemblies also included more contigs that matched both mouse and *S. mansoni* transcripts (i.e., potential synthetic chimeras; Table S8; Figure 1(b)).

Supplementary Tables

Supplementary Table S1. Transcriptome assembly. Transcriptome assembly was accomplished by using both de novo (Programs: Trinity de novo and SOAPdenovo–Trans) and reference guided (Programs: Genome guided Trinity, Cufflinks) assembly approaches. Four assemblies using: (i) uninfected mice (reads U1, U2); (ii) low–infected mice (reads L1, L2); (iii) high infected mice (reads H1, H2); and all mice (reads U1, U2, L1, L2, H1, H2), were produced from each program.

*A separate set of Genome–guided Trinity assemblies were made using *S. mansoni* as the reference genome.

Assembler name	Assembly procedure
Trinity *de novo*	No reference
SOAPdenovo-Trans	No reference, multiple k-mer approach (k-mers 21, 25, 29, 33, 37) follwed by combining assemblies using cd-hit-est
Genome guided Trinity (Trinity GG)	GSNAP aligner, *Mus musculus* reference genome, **S. mansoni* reference genome
Cufflink	*Mus musculus* reference genome, bowtie-Tophat-cufflink pipeline

Supplementary Table S2. Primer sequences developed for the analysis. PCR profile used was 94˚C 5 minutes, 40 cycles of 94˚C for 45s, 30s at annealing temperature, 30s at 68˚C followed by 5 minutes at 72˚C

Results analyzed using Nanodrop 8000, 1% Agarose gel.
*: non-target pcr primers
ɸ: qPCR primers (differential gene expression analysis). Swissprot gene name and accession numbers are given in target and description columns.

Target	Description	Sequence (5'- 3')	Tm (˚C)	Amplicon length (bp)
Contig 437 *	SOAPdenovo-Trans contig	F: TTGCATTGACCCACACAGTT	55.2	240
		R: TAATGGACAGGTTGGGTGGT		
Contig c92104 *	Trinity denovo contig	F: GAAAAGACCCACGCATCATT	53.5	194
		R: TGGGGAGTGTGAGAGTGAGC		
Contig c93270 *	Trinity denovo contig	F: CATCGGTCTTCTGCCAAAAT	53.3	215
		R: GACGGAGCTATGGAGGTCAG		
Scaffold 152316 *	SOAPdenovo-Trans scaffold	F: CTCAAAACAGCCAATCAACG	52.5	223
		R: GATGGCTGCCCATAGTTCAT		
Scaffold 296945 *	SOAPdenovo-Trans scaffold	F: CACTGGGTCCACTGGAACTT	57.1	164
		R: TGAACGAGTCTGGTCAGGTG		
Contig 18347 *	SOAPdenovo-Trans contig	F: CCGGATTTCGTGAAGTCATC	53.5	161
		R: TCACTCTCACCACCATGGAC		
A6H584 ɸ	Gene: CO6A5_MOUSE (swissprot), up regulated	F: CAGACGTGGTGTTTCTGGTG	59	167
		R: CAGCTGGAACTCATTGTGGA		
P48298 ɸ	Gene: CCL11_MOUSE (swissprot), up regulated	F: CACGGTCACTTCCTTCACCT	59	152
		R: CTATGGCTTTCAGGGTGCAT		
P01868 ɸ	Gene: IGHG1_MOUSE (swissprot), up regulated	F: CAAGAACACTCAGCCCATCA	59	156
		R: TGGGAGAGGCTCTTCTCAGT		
P35459 ɸ	Gene: LY6D_MOUSE (swissprot), up regulated	F: GTCTGCCCGTCCAACTTCTA	59	167
		R: CATAGGTCAGTCTGGCAGCA		
P40936 ɸ	Gene: INMT_MOUSE (swissprot), down regulated	F: AGCCTGCAGAACCTCTACCA	59	193

		R: AGGCTCCTGGCTCTTTCTTC		
Q8BWU8 ᶲ	Gene: AT2L1_MOUSE (swissprot), down regulated	F: CCTCCACGACAACATCATTG<br>R: GGTCAAGGGTGATCACATCC	59	161
Q6PFC5 ᶲ	Gene: LRIT2_MOUSE (swissprot), down regulated	F: GCTTGGTTGTGGAGCTGAGT<br>R: AGAGACAGGACCCTGGCATA	59	163
Q7TMF5 ᶲ	Gene: SPA12_MOUSE (swissprot), down regulated	F: CTCTCCAGTCAGGGTCCAAG<br>R: AGGCAGTGGAGATGCTCAGT	59	155
Q14DH7 ᶲ	Gene: ACSS3_MOUSE (swissprot), down regulated	F: GGTTGCAATGTCGCAAAGTA<br>R: TGCGAAGTGGGTCTTGTACTC	59	173
O35949 ᶲ	Gene: ELOV3_MOUSE (swissprot), down regulated	F: AGGCCCTTTTTGGAGGAGTA<br>R: ATCCGTGTAGATGGCAAAGC	59	249

Supplementary Table S3. RNA quality**.** RNA Integrity Number (RIN) was used to assert the quality of RNA samples used in cDNA library construction. Gene expression assays are sensitive to RNA quality, and Romero et al. (2014) recommended RNA integrity numbers (RIN) >7.9 and <1 difference in RIN value between samples. The quality of our RNA samples (≥8.6 RIN; ≤0.5 difference between samples) exceeded their recommendations, which suggests that degradation did not compromise our experiments.
U1, U2: Uninfected livers; L1, L2: Low–infected livers; H1, H2: High infected livers

Sample	RIN
U1	8.6
U2	9
L1	9
L2	9.1
H1	9
H2	8.8

Reference: Romero IG , Pai1 AA, Tung J, Gilad Y. 2014. RNA-seq: impact of RNA degradation on transcript quantification. BMC Biology

12:42

Supplementary Table S4. Descriptive statistics of raw reads. Average read length is 100 and N50 is 101.

* Trimmomatic discarded 1–2% of each library due to low quality.

# Represent concordant matches (BLASTN %ID >90%; E–value=10–12)

Library	Number of reads		Percentage GC content	Percentage of reads matching to *S. mansoni* transcriptome #
	Before filtering	After filtering *		
U1	66,852,914	65,914,556	47.2	0.0001370
U2	65,877,464	65,036,006	47.25	0.0001570
L1	59,720,992	58,929,048	47.75	0.093840
L2	62,428,548	61,653,850	48.05	0.0057970
H1	62,094,698	61,448,830	48.05	0.0254910
H2	66,793,630	66,144,488	47.95	0.0251030

Supplementary Table S5: Descriptive statistics of transcriptome assemblies. All four assemblers produced the greatest number of contigs when all samples (L1, L2, H1, H2, U1, U2) were combined. SOAPdenovo–Trans and Cufflinks produced the largest contigs, but SOAPdenovo–Trans contigs contained many gaps in the scaffolds compared to Cufflinks. Reference guided assemblies yielded higher N50 and N50 ratios, suggesting the presence of longer/complete transcripts compared to *de novo* assemblies. In both *de novo* and reference guided assemblies, a slight decrease in N50 is apparent in highly infected liver transcriptomes, possibly due to the presence of more short contigs. All four approaches produced assemblies that contained >89% of CEG genes

De novo assemblies: ☐

Reference guided assemblies: ☐ (grey)

* N50 ratio represents: N50 of assembly/ N50 of Ensemble *Mus musculus* mRNA library

All statistics are based on sequences of size ≥ 500 bp, unless otherwise noted

Statistics	Trinity *de novo* U1U2	Trinity *de novo* L1L2	Trinity *de novo* H1H2	Trinity *de novo* all	SOAPdenovo -*Trans* U1U2	SOAPdenovo -Trans L1L2	SOAPdenovo -Trans H1H2	SOAPdenovo -Trans all
No of contigs (>0 bp)	113238	123682	147683	231862	108084	114001	262174	229097
No of contigs (>1000 bp)	27645	30296	33973	47241	34741	37174	21853	74307
Total length (>0 bp)	104788136	114716272	130993616	191023307	131504041	138081451	105695266	282696488
Total length (>1000 bp)	71431814	78281457	86429014	117593714	107535591	113017862	53505770	228829309
No of contigs (>500 bp)	47218	51675	60178	92130	51945	55101	38933	114914
Largest contig	19635	18202	16919	26101	26365	26618	15700	33066
Total length (>500 bp)	85057486	93156478	104567476	148633327	119817534	125782377	65402223	257878247
GC (%)	47.87	48.8	47.98	47.51	46.41	46.69	47.05	46.05
N50	2600	2603	2511	2315	3604	3472	2374	3512
**N50 ratio ***	**0.819414**	**0.820359**	**0.791365**	**0.729593**	**1.135834**	**1.094233**	**0.748188**	**1.106839**
N75	1384	1389	1317	1143	1877	1859	1259	1764
L50	10006	11010	12758	18591	10461	11340	8467	21856
L75	21142	23155	27012	41514	21936	23609	17876	47698
Ns per 100 kbp	0	0	0	0	2365.28	2330.55	857.26	2819.41
Percentage of 248 ultra-conserved CEGs present								
Complete	97.98	98.79	98.79	99.19	98.79	95.56	95.16	99.6
Partial	99.19	100	100	100	99.6	97.58	98.79	100

Statistics	Trinity GG U1U2	Trinity GG L1L2	Trinity GG H1H2	Trinity GG all	Cufflink U1U2	Cufflink L1L2	Cufflink H1H2	Cufflink all
No of contigs (>0 bp)	138219	151410	178251	276730	25039	27120	30128	38852
No of contigs (>1000 bp)	30278	33095	37477	55060	21165	23258	25564	34477
Total length (>0 bp)	123960109	134819502	155057309	232101194	73544770	79846302	87733710	131889705
Total length (>1000 bp)	82765086	89569762	100614691	144993836	70971532	77274726	84687431	128944001
No of contigs (>500 bp)	53172	58461	68296	106567	24075	26151	28981	37796
Largest contig	19635	18107	22387	23538	27706	26369	26425	28019
Total length (>500 bp)	98571586	107040204	121833137	180533176	73193717	79499740	87316520	131507969
GC (%)	47.44	47.71	47.58	46.93	47.73	47.92	47.89	47.7
N50	2805	2761	2690	2534	4047	3980	3935	4555
**N50 ratio ***	**0.884021**	**0.870154**	**0.847778**	**0.798613**	**1.27545**	**1.25433**	**1.24015**	**1.43555**
N75	1418	1400	1342	1204	2487	2488	2486	2909
L50	10592	11743	13616	20332	5928	6562	7330	9562
L75	22848	25231	29505	46301	11656	12850	14274	18549
Ns per 100 kbp	0	0	0	0	0.01	0.01	0.12	0.08
Percentage of 248 ultra-conserved CEGs present								
Complete	98.79	99.19	98.39	98.39	89.92	90.73	89.52	92.74
Partial	99.6	100	100	100	95.16	95.16	93.55	95.97

Supplementary Table S6. Structural errors in transcriptomes**.** Structural errors in transcriptome assembly compared to the Ensembl *Mus musculus* cDNA data set. Both de novo Trinity and Cufflinks assemblies exhibited an increase in breakpoints, relocations, translocations, insertions and indels as parasite load increased (e.g., the SOAPdenovo–Trans H1H2 assembly contains many inversions compared to the other assemblies).

Assembly	Number of aligned sequences	Number of aligned bases	% Aligned sequences	% Aligned bases	1-to-1 Alignments	M-to-M Alignments	Breakpoints	Relocations	Translocations	Inversions	Insertions	Indels
Trinity *de novo* U1U2	46444	67433633	41.01	64.35	41069	143940	**187547**	**14**	**3174**	0	**57104**	**32683**
Trinity *de novo* L1L2	50345	74595519	40.71	65.03	44173	152786	**202236**	**21**	**3465**	1	**63955**	**36789**
Trinity *de novo* H1H2	55080	80681885	37.30	61.59	47354	162395	**217220**	**28**	**3719**	1	**74365**	**39850**
Trinity *de novo* all	63861	94227571	27.54	49.33	53569	178687	**248167**	**29**	**4459**	1	**97581**	**44759**
SOAPdenovo-*Trans* U1U2	46293	76412846	42.83	58.11	35104	156749	219131	21	3535	1	84964	90414
SOAPdenovo-Trans L1L2	49504	82702350	43.42	59.89	37480	165549	232147	26	3896	1	90697	96916
SOAPdenovo-Trans H1H2	72510	64626424	28.08	55.06	59472	192689	199484	23	3615	205	71118	66070
SOAPdenovo-Trans all	74250	121263720	32.41	42.90	48480	220318	331736	27	5349	2	178736	1E+05
Trinity GG U1U2	68882	80187625	49.84	64.69	51209	258703	345807	12	4432	3	217545	55787
Trinity GG L1L2	74673	88104474	49.32	65.35	39538	181193	230082	24	3024	2	108745	40106
Trinity GG H1H2	83303	96649941	46.73	62.33	42736	193041	248637	22	3313	2	119817	44208
Trinity GG all	111818	119116050	40.41	51.32	45531	209563	272272	16	3559	6	141865	48967
Cufflink U1U2	22794	59882120	91.03	81.42	21129	106781	**16468**	**4**	**3426**	1	**39123**	**15239**
Cufflink L1L2	24852	65944427	91.64	82.59	22908	113676	**175192**	**6**	**3698**	1	**42106**	**16397**
Cufflink H1H2	27327	71238314	90.70	81.20	24600	121094	**187656**	**5**	**3950**	1	**49869**	**17220**
Cufflink all	35457	103910826	91.26	78.79	26249	144795	**234781**	**6**	**4097**	1	**90122**	**18201**

Supplementary Table S7. Average Ortholog hit ratio (OHR) of transcriptomes. OHR was calculated separately using swissprot *Mus musculus* and *S. mansoni* protein sequences.

Assembly	Compared to *Mus musculus* proteins				Compared to *S. mansoni* proteins			
	ohr >1	ohr 0.8-1	ohr < 0.8	unique hits	ohr >1	ohr 0.8-1	ohr < 0.8	unique hits
Trinity *de novo* U1U2	1.33	57.82	40.85	11346	5.41	35.14	59.46	74
Trinity *de novo* L1L2	1.35	58.58	40.08	12192	5.06	31.65	63.29	79
Trinity *de novo* H1H2	1.30	55.33	**43.37**	12646	3.75	30.00	66.25	80
Trinity *de novo* all	3.95	72.33	23.71	13001	8.54	39.02	52.44	82
SOAPdenovo-*Trans* U1U2	2.71	74.06	23.23	10962	16.00	57.33	26.67	75
SOAPdenovo-Trans L1L2	2.57	76.42	21.01	11790	18.42	55.26	26.32	76
SOAPdenovo-Trans H1H2	1.59	60.96	**37.46**	12284	13.92	51.90	34.18	79
SOAPdenovo-Trans all	3.18	76.25	20.57	12713	18.75	58.75	22.50	80
Trinity GG U1U2	3.47	70.82	25.71	11376	18.92	55.41	25.68	74
Trinity GG L1L2	3.50	72.97	23.53	12235	17.95	52.56	29.49	78
Trinity GG H1H2	3.80	72.69	23.51	12648	16.67	28.21	53.85	78
Trinity GG all	4.20	74.16	21.64	13043	17.95	53.85	28.21	78
Cufflink U1U2	1.39	66.57	32.04	10099	6.67	32.00	61.33	75
Cufflink L1L2	1.42	67.64	30.94	11023	3.90	40.26	55.84	77
Cufflink H1H2	1.35	65.72	32.93	11470	6.41	33.33	60.26	78
Cufflink all	1.46	65.95	32.59	11630	5.06	34.18	60.76	79

Supplementary Table S8. Assembly annotation statistics. Annotation was done using BLASTX against swissprot protein database. 1000 hits were collected per query (E–value: 10–6; Percentage identity ≥90%) and then separated as chordate and non–chordate hits.

Assembly	Total Number of contigs with hits	Percentage of contigs with all chordate hits	Percentage of contigs with both chordate and non-chordate hits	Percentage of contigs with all non-chordate hits
Trinity *de novo* U1U2	25722	99.22	0.73	0.05
Trinity *de novo* L1L2	28226	99.07	0.80	0.13
Trinity *de novo* H1H2	30534	99.03	0.74	0.23
Trinity *de novo* all	33007	98.95	0.79	0.25
SOAPdenovo-*Trans* U1U2	22779	98.60	1.21	0.18
SOAPdenovo-Trans L1L2	24355	98.49	1.30	0.21
SOAPdenovo-Trans H1H2	29413	98.20	1.34	0.47
SOAPdenovo-Trans all	13299	98.40	1.18	0.41
Trinity GG U1U2	18550	89.65	3.09	7.26
Trinity GG L1L2	39146	89.95	2.54	7.51
Trinity GG H1H2	42487	89.71	2.66	7.63
Trinity GG all	52051	88.63	3.08	8.29
Cufflink U1U2	18805	99.07	0.91	0.02
Cufflink L1L2	20570	99.09	0.90	0.01
Cufflink H1H2	22393	99.09	0.88	0.03
Cufflink all	29070	99.14	0.85	0.01

Supplementary Table S9. Comparing assemblies against mouse and *S. mansoni* transcriptomes to identify non-targets. Comparison was done using BLASTN (E–value: 10–6; Percentage identity ≥90%)

Assembly	Total Number of contigs with hits	Percentage of contigs with all mouse hits	Percentage of contigs with both mouse and *S. mansoni* hits/ possible chimera	Percentage of contigs with all *S. mansoni* hits
Trinity *de novo* U1U2	53807	99.98	0.01	0.00
Trinity *de novo* L1L2	40582	99.07	0.47	0.46
Trinity *de novo* H1H2	43776	96.23	1.89	1.88
Trinity *de novo* all	48047	95.50	2.25	2.25
SOAPdenovo-*Trans* U1U2	53483	99.98	0.02	0.00
SOAPdenovo-Trans L1L2	57393	99.54	0.24	0.22
SOAPdenovo-Trans H1H2	81992	95.99	2.01	2.00
SOAPdenovo-Trans all	102048	98.29	0.86	0.85
Trinity GG U1U2	22972	99.96	0.03	0.01
Trinity GG L1L2	24986	99.98	0.02	0.00
Trinity GG H1H2	27368	99.98	0.02	0.00
Trinity GG all	35665	99.90	0.07	0.03
Cufflink U1U2	62310	99.99	0.01	0.00
Cufflink L1L2	67034	99.98	0.02	0.00
Cufflink H1H2	75143	99.98	0.02	0.00
Cufflink all	103090	99.98	0.02	0.00

Supplementary Table S10. Comparison of predicted ORFs against mouse and *S. mansoni* transcriptomes to identify sequences of non-target origin (BLASTN; E–value: 10^{-6}; Percentage identity ≥90%).

Assembly	All predicted ORFs	ORFs with BLASTP hits	Percentage of ORFs with all mouse hits	Percentage of ORFs with both mouse and *S. mansoni* hits/ possible chimera	Percentage of ORFs with all *S. mansoni* hits
Trinity *de novo* U1U2	34893	31223	99.99	0.01	0.00
Trinity *de novo* L1L2	38926	34870	99.81	0.00	0.19
Trinity *de novo* H1H2	43399	38227	99.13	0.00	0.87
Trinity *de novo* all	53651	43624	99.00	0.00	1.00
SOAPdenovo-*Trans* U1U2	9413	7571	100.00	0.00	0.00
SOAPdenovo-Trans L1L2	10150	8136	99.98	0.00	0.02
SOAPdenovo-Trans H1H2	6661	5774	99.81	0.03	0.16
SOAPdenovo-Trans all	73339	52329	99.43	0.01	0.56
Trinity GG U1U2	44336	36845	99.98	0.02	0.00
Trinity GG L1L2	48642	40455	100.01	0.01	0.00
Trinity GG H1H2	54952	45065	99.99	0.01	0.00
Trinity GG all	72122	54078	99.96	0.03	0.01
Cufflink U1U2	26936	25238	99.99	0.01	0.00
Cufflink L1L2	29693	27972	100.00	0.00	0.00
Cufflink H1H2	32575	30481	100.00	0.00	0.00
Cufflink all	45938	42504	100.00	0.00	0.00

Supplementary Table S11. Annotation statistics of *S. mansoni*–guided Trinity assemblies. Annotation was performed using BLASTX against the Swissprot database. 1000 hits were collected per query (E–value: 10–6; Percentage identity ≥90%) and then separated as chordate and non–chordate hits.

Assembly	Total number of contigs	Total number of contigs with hits	Percentage of contigs with all chordate hits	Percentage of contigs with both chordate and non-chordate hits	Percentage of contigs with all non-chordate hits
Trinity GG U1U2	64	7.00	100.00	0.00	0.00
Trinity GG L1L2	333	53.00	11.32	41.51	47.17
Trinity GG H1H2	1343	90.00	10.00	35.56	54.44
Trinity GG all	1850	108.00	8.33	29.63	62.04

Supplementary Table S12. BLASTN matches of *S. mansoni*–guided Trinity assemblies. Comparisons was done against mouse and *S. mansoni* transcriptomes to identify the possible origin. Comparison was done using BLASTN (E–value: 10^{-6}; identity ≥90%).

Assembly	total number of contigs with hits	Percentage of contigs with all mouse hits	Percentage of contigs with both mouse and *S. mansoni* hits	Percentage of contigs with all *S. mansoni* hits
Trinity GG U1U2	59	79.66	20.34	0.00
Trinity GG L1L2	305	21.31	4.26	74.43
Trinity GG H1H2	1160	5.60	1.29	93.10
Trinity GG all	1166	6.09	1.37	92.54

Supplementary Table S13. Number of significant GO terms and KEGG IDs corresponding to DE genes, before and after filtering out *S. mansoni* reads from mouse liver transcriptomes.

Assembly type	UH		UL		LH	
	Up regulated	Down regulated	Up regulated	Down regulated	Up regulated	Down regulated
(a) Significant GO terms						
With non-target contigs	465	281	411	216	79	174
Without non-target contigs	451	284	392	216	78	173
(b) Significant KEGG IDs						
With non-target contigs	43	37	29	37	5	19
Without non-target contigs	41	37	29	34	5	20

Supplementary Table S14. Representation of GO term categories in significantly expressed genes.
UH: U vs H; UL: U vs L; LH: L vs H
U: Uninfected; L: Low–infected; H: High–infected

GO category	UH		UL		LH	
	Up	Down	Up	Down	Up	Down
Biological process	78%	51%	78%	45%	71%	52%
Cellular Component	11%	19%	8%	23%	19%	20%
Molecular Function	12%	30%	13%	31%	10%	28%

Supplementary Table S15. DE (upregulated in L compared to U) genes in GO term category immune system process.

ID	Gene Name
P11928	2'-5' oligoadenylate synthetase 1A
Q8VI93	2'-5' oligoadenylate synthetase 3
Q8VI94	2'-5' oligoadenylate synthetase-like 1
Q9Z2F2	2'-5' oligoadenylate synthetase-like 2
Q7TSA3	B and T lymphocyte associated
Q07440	B-cell leukemia/lymphoma 2 related protein A1a
Q6QLQ4	C-type lectin domain family 7, member a
P10810	CD14 antigen
Q62192	CD180 antigen
P04235	CD3 antigen, delta polypeptide
Q3U497	CD300 antigen like family member B
Q6SJQ0	CD300A antigen
Q8K249	CD300e antigen
P27512	CD40 antigen
P13379	CD5 antigen
P04441	CD74 antigen (invariant polypeptide of major histocompatibility complex, class II antigen-associated)
P01731	CD8 antigen, alpha chain
P10300	CD8 antigen, beta chain 1
Q00609	CD80 antigen
P42082	CD86 antigen
O35601	FYN binding protein
P20491	Fc receptor, IgE, high affinity I, gamma polypeptide
P26151	Fc receptor, IgG, high affinity I
P08508	Fc receptor, IgG, low affinity III
Q03267	IKAROS family zinc finger 1
Q8R4B8	NLR family, pyrin domain containing 3
P17433	SFFV proviral integration 1

P01849	T-cell receptor alpha chain
Q7TSN2	cDNA sequence AF251705
Q9QZM3	cardiotrophin-like cytokine factor 1
Q8CIS0	caspase recruitment domain family, member 11
A2AIV8	caspase recruitment domain family, member 9
P48298	chemokine (C-C motif) ligand 11
P10148	chemokine (C-C motif) ligand 2
Q9JKC0	chemokine (C-C motif) ligand 24
P14097	chemokine (C-C motif) ligand 4
P27784	chemokine (C-C motif) ligand 6
Q03366	chemokine (C-C motif) ligand 7
Q9Z121	chemokine (C-C motif) ligand 8
P51683	chemokine (C-C motif) receptor 2
P51682	chemokine (C-C motif) receptor 5
P70658	chemokine (C-X-C motif) receptor 4
P79621	class II transactivator
P14106	complement component 1, q subcomponent, beta polypeptide
Q8C3J5	dedicator of cyto-kinesis 2
Q00612	glucose-6-phosphate dehydrogenase X-linked
Q64281	glycoprotein 49 A; leukocyte immunoglobulin-like receptor, subfamily B, member 4
Q9Z0E6	guanylate binding protein 2
Q61107	guanylate binding protein 3
P49710	hematopoietic cell specific Lyn substrate 1
P01902	histocompatibility 2, K1, K region; similar to H-2K(d) antigen
P04228	histocompatibility 2, class II antigen A, alpha; histocompatibility 2, class II antigen E alpha
P01921	histocompatibility 2, class II antigen A, beta 1; response to metastatic cancers 2; similar to H-2 class II histocompatibility antigen, A-D beta chain precursor
P01915	histocompatibility 2, class II antigen E beta
P28078	histocompatibility 2, class II, locus DMa
P35737	histocompatibility 2, class II, locus Mb1

Q9BDB7	histocompatibility 28
Q99N13	histone deacetylase 9
Q9JHJ8	icos ligand
Q60766	immunity-related GTPase family M member 1
P01872	immunoglobulin heavy chain 6 (heavy chain of IgM)
P01868	immunoglobulin heavy constant gamma 1 (G1m marker)
P05555	integrin alpha M
Q9QXH4	integrin alpha X
P13597	intercellular adhesion molecule 1
P70434	interferon regulatory factor 7
P23611	interferon regulatory factor 8
P01582	interleukin 1 alpha
Q9Z2B1	interleukin 18 receptor accessory protein
O54709	killer cell lectin-like receptor subfamily K, member 1
Q9JHL0	linker for activation of T cells family, member 2
Q60787	lymphocyte cytosolic protein 2
Q8BHB3	lymphocyte transmembrane adaptor 1
P09922	myxovirus (influenza virus) resistance 1
Q09014	neutrophil cytosolic factor 1
O35904	phosphatidylinositol 3-kinase catalytic delta polypeptide; RIKEN cDNA 2610208K16 gene
Q8CIH5	phospholipase C, gamma 2
Q8BVK9	predicted gene 15753; Sp110 nuclear body protein
P40223	predicted gene 4223; similar to Csf3r protein; colony stimulating factor 3 receptor (granulocyte)
Q9Z2U4	predicted gene 9907
P28063	proteasome (prosome, macropain) subunit, beta type 8 (large multifunctional peptidase 7)
O35522	proteasome (prosome, macropain) subunit, beta type 9 (large multifunctional peptidase 2)
Q64695	protein C receptor, endothelial
P29352	protein tyrosine phosphatase, non-receptor type 22 (lymphoid)
P06800	protein tyrosine phosphatase, receptor type, C
Q9D3G9	ras homolog gene family, member H

Q03347	runt related transcription factor 1
Q62170	selectin, platelet (p-selectin) ligand
Q5I2A0	serine (or cysteine) peptidase inhibitor, clade A, member 3G
P15702	sialophorin
P97484	similar to Leukocyte immunoglobulin-like receptor, subfamily B (with TM and ITIM domains), member 3; leukocyte immunoglobulin-like receptor, subfamily B (with TM and ITIM domains), member 3
Q8VCH2	similar to RIKEN cDNA 4732429D16 gene; RIKEN cDNA 4732429D16 gene
Q9JHH5	similar to Small inducible cytokine B11 precursor (CXCL11) (Interferon-inducible T-cell alpha chemoattractant) (I-TAC); chemokine (C-X-C motif) ligand 11
Q01514	similar to guanylate nucleotide binding protein 1; guanylate binding protein 1
P41251	solute carrier family 11 (proton-coupled divalent metal ion transporters), member 1
Q60611	special AT-rich sequence binding protein 1
P01831	thymus cell antigen 1, theta
Q9EPQ1	toll-like receptor 1
Q6R5P0	toll-like receptor 11
Q6R5N8	toll-like receptor 13
Q9QUN7	toll-like receptor 2
Q9QUK6	toll-like receptor 4
P58681	toll-like receptor 7
P58682	toll-like receptor 8
Q9JJ11	transforming, acidic coiled-coil containing protein 3
Q3TBT3	transmembrane protein 173
P21958	transporter 1, ATP-binding cassette, sub-family B (MDR/TAP)
B2RUP2	unc-13 homolog D (C. elegans)
P27870	vav 1 oncogene

Supplementary Table S16. DE (upregulated in H compared to U) genes in GO term category immune system process.

ID	Gene Name
P11928	2'-5' oligoadenylate synthetase 1A
Q8VI93	2'-5' oligoadenylate synthetase 3
Q8VI94	2'-5' oligoadenylate synthetase-like 1
Q9Z2F2	2'-5' oligoadenylate synthetase-like 2
Q7TSA3	B and T lymphocyte associated
Q07440	B-cell leukemia/lymphoma 2 related protein A1a
Q9QUN3	B-cell linker
O54918	BCL2-like 11 (apoptosis facilitator)
Q9WVF9	C-type lectin domain family 2, member i
Q9Z2H6	C-type lectin domain family 4, member d
Q6QLQ4	C-type lectin domain family 7, member a
P10810	CD14 antigen
Q62192	CD180 antigen
P25918	CD19 antigen
P24807	CD24a antigen
P41272	CD27 antigen
P04235	CD3 antigen, delta polypeptide
Q3U497	CD300 antigen like family member B
Q6SJQ0	CD300A antigen
Q8K249	CD300e antigen
P27512	CD40 antigen
P13379	CD5 antigen
P04441	CD74 antigen (invariant polypeptide of major histocompatibility complex, class II antigen-associated)
P11911	CD79A antigen (immunoglobulin-associated alpha)
P01731	CD8 antigen, alpha chain
P10300	CD8 antigen, beta chain 1
Q00609	CD80 antigen

P42082	CD86 antigen
Q00342	FMS-like tyrosine kinase 3
O35601	FYN binding protein
P41047	Fas ligand (TNF superfamily, member 6)
P20491	Fc receptor, IgE, high affinity I, gamma polypeptide
P26151	Fc receptor, IgG, high affinity I
P08508	Fc receptor, IgG, low affinity III
Q03267	IKAROS family zinc finger 1
Q62137	Janus kinase 3
Q5DU56	NLR family, CARD domain containing 3
Q8R4B8	NLR family, pyrin domain containing 3
P17433	SFFV proviral integration 1
Q9JID9	SH2B adaptor protein 2
Q06831	SRY-box containing gene 19; SRY-box containing gene 4
Q8R4L0	Src-like-adaptor 2
P01849	T-cell receptor alpha chain
P03958	adenosine deaminase
Q7TSN2	cDNA sequence AF251705
P54285	calcium channel, voltage-dependent, beta 3 subunit
Q9QZM3	cardiotrophin-like cytokine factor 1
Q8CIS0	caspase recruitment domain family, member 11
A2AIV8	caspase recruitment domain family, member 9
P48298	chemokine (C-C motif) ligand 11
Q62401	chemokine (C-C motif) ligand 12; similar to monocyte chemoattractant protein-5
P10148	chemokine (C-C motif) ligand 2
Q9JKC0	chemokine (C-C motif) ligand 24
P10855	chemokine (C-C motif) ligand 3
P14097	chemokine (C-C motif) ligand 4
P27784	chemokine (C-C motif) ligand 6
Q03366	chemokine (C-C motif) ligand 7

Q9Z121	chemokine (C-C motif) ligand 8
P51670	chemokine (C-C motif) ligand 9
P51683	chemokine (C-C motif) receptor 2
P51682	chemokine (C-C motif) receptor 5
P47774	chemokine (C-C motif) receptor 7
P12850	chemokine (C-X-C motif) ligand 1
P10889	chemokine (C-X-C motif) ligand 2
P70658	chemokine (C-X-C motif) receptor 4
O35188	chemokine (C-X3-C motif) ligand 1
P79621	class II transactivator
P14106	complement component 1, q subcomponent, beta polypeptide
P30993	complement component 5a receptor 1
P09793	cytotoxic T-lymphocyte-associated protein 4; similar to cytotoxic T-lymphocyte associated molecule 4
Q8C3J5	dedicator of cyto-kinesis 2
P56386	defensin beta 1
Q922Y0	dual-specificity tyrosine-(Y)-phosphorylation regulated kinase 3
O54839	eomesodermin homolog (Xenopus laevis)
Q00612	glucose-6-phosphate dehydrogenase X-linked
Q64281	glycoprotein 49 A; leukocyte immunoglobulin-like receptor, subfamily B, member 4
Q9Z0E6	guanylate binding protein 2
Q61107	guanylate binding protein 3
Q6VYH9	hematopoietic SH2 domain containing
P49710	hematopoietic cell specific Lyn substrate 1
P01902	histocompatibility 2, K1, K region; similar to H-2K(d) antigen
P04228	histocompatibility 2, class II antigen A, alpha; histocompatibility 2, class II antigen E alpha
P01921	histocompatibility 2, class II antigen A, beta 1; response to metastatic cancers 2; similar to H-2 class II histocompatibility antigen, A-D beta chain precursor
P01915	histocompatibility 2, class II antigen E beta
P28078	histocompatibility 2, class II, locus DMa
P35737	histocompatibility 2, class II, locus Mb1
Q9BDB7	histocompatibility 28

Q8C2B3	histone deacetylase 7; similar to histone deacetylase 7A
Q99N13	histone deacetylase 9
P02831	homeo box A3
P09026	homeo box B3
Q9JHJ8	icos ligand
Q60766	immunity-related GTPase family M member 1
P01863	immunoglobulin heavy chain 3 (serum IgG2b); Immunoglobulin heavy chain (gamma polypeptide)
P01872	immunoglobulin heavy chain 6 (heavy chain of IgM)
P01868	immunoglobulin heavy constant gamma 1 (G1m marker)
P01592	immunoglobulin joining chain
Q6P549	inositol polyphosphate phosphatase-like 1
Q61739	integrin alpha 6
P05555	integrin alpha M
Q9QXH4	integrin alpha X
P13597	intercellular adhesion molecule 1
P15314	interferon regulatory factor 1
Q64287	interferon regulatory factor 4
P70434	interferon regulatory factor 7
P23611	interferon regulatory factor 8
P01582	interleukin 1 alpha
P18893	interleukin 10
O54824	interleukin 16
Q9Z2B1	interleukin 18 receptor accessory protein
P01590	interleukin 2 receptor, alpha chain
P16872	interleukin 7 receptor
O54709	killer cell lectin-like receptor subfamily K, member 1
Q9JHL0	linker for activation of T cells family, member 2
Q60787	lymphocyte cytosolic protein 2
Q8BHB3	lymphocyte transmembrane adaptor 1
P19437	membrane-spanning 4-domains, subfamily A, member 1

P09922	myxovirus (influenza virus) resistance 1
Q09014	neutrophil cytosolic factor 1
Q8K3Z0	nucleotide-binding oligomerization domain containing 2
O88593	peptidoglycan recognition protein 1; similar to peptidoglycan recognition protein
O35904	phosphatidylinositol 3-kinase catalytic delta polypeptide; RIKEN cDNA 2610208K16 gene
Q8CIH5	phospholipase C, gamma 2
Q9Z126	platelet factor 4
Q8BVK9	predicted gene 15753; Sp110 nuclear body protein
P10417	predicted gene 3655; B-cell leukemia/lymphoma 2
P40223	predicted gene 4223; similar to Csf3r protein; colony stimulating factor 3 receptor (granulocyte)
P01758, P06336	predicted gene 5353; immunoglobulin heavy chain (J558 family); similar to Ig heavy chain V region 108A precursor; similar to Ig heavy chain V-I region V35 precursor; predicted gene 900; immunoglobulin heavy chain complex; similar to Ig H-chain V-JH1-region; immunoglobulin heavy variable V1-31; immunoglobulin heavy chain 2 (serum IgA)
Q9Z2U4	predicted gene 9907
P28063	proteasome (prosome, macropain) subunit, beta type 8 (large multifunctional peptidase 7)
O35522	proteasome (prosome, macropain) subunit, beta type 9 (large multifunctional peptidase 2)
Q64695	protein C receptor, endothelial
P28867	protein kinase C, delta
P29352	protein tyrosine phosphatase, non-receptor type 22 (lymphoid)
P06800	protein tyrosine phosphatase, receptor type, C
Q9D3G9	ras homolog gene family, member H
Q03347	runt related transcription factor 1
Q62170	selectin, platelet (p-selectin) ligand
Q5I2A0	serine (or cysteine) peptidase inhibitor, clade A, member 3G
P15702	sialophorin
P97484	similar to Leukocyte immunoglobulin-like receptor, subfamily B (with TM and ITIM domains), member 3; leukocyte immunoglobulin-like receptor, subfamily B (with TM and ITIM domains), member 3
Q8VCH2	similar to RIKEN cDNA 4732429D16 gene; RIKEN cDNA 4732429D16 gene
P01863	similar to gamma-2a immunoglobulin heavy chain; immunoglobulin heavy chain 1a (serum IgG2a); immunoglobulin heavy chain 1b (serum IgG2c)
Q01514	similar to guanylate nucleotide binding protein 1; guanylate binding protein 1
P41251	solute carrier family 11 (proton-coupled divalent metal ion transporters), member 1

Q60611	special AT-rich sequence binding protein 1
Q3UUV5	src family associated phosphoprotein 1
P01831	thymus cell antigen 1, theta
Q9EPQ1	toll-like receptor 1
Q6R5P0	toll-like receptor 11
Q6R5N8	toll-like receptor 13
Q9QUN7	toll-like receptor 2
Q9QUK6	toll-like receptor 4
Q9EPW9	toll-like receptor 6
P58681	toll-like receptor 7
P58682	toll-like receptor 8
Q9EQU3	toll-like receptor 9
P04202	transforming growth factor, beta 1
Q62312	transforming growth factor, beta receptor II
Q9JJ11	transforming, acidic coiled-coil containing protein 3
Q3TBT3	transmembrane protein 173
P21958	transporter 1, ATP-binding cassette, sub-family B (MDR/TAP)
Q9WU72	tumor necrosis factor (ligand) superfamily, member 13b
B2RUP2	unc-13 homolog D (C. elegans)
P27870	vav 1 oncogene

Supplementary Table S17. DE (upregulated H compared to L) genes in GO term category antigen binding.

ID	Gene Name
P01872	immunoglobulin heavy chain 6 (heavy chain of IgM)
P01868	immunoglobulin heavy constant gamma 1 (G1m marker)
P01592	immunoglobulin joining chain
P01723	immunoglobulin lambda chain, constant region 2; immunoglobulin lambda chain, constant region 1; immunoglobulin lambda chain, variable 1
P06336	predicted gene 5353; immunoglobulin heavy chain (J558 family); similar to Ig heavy chain V region 108A precursor; similar to Ig heavy chain V-I region V35 precursor; predicted gene 900; immunoglobulin heavy chain complex; similar to Ig H-chain V-JH1-region; immunoglobulin heavy variable V1-31; immunoglobulin heavy chain 2 (serum IgA)
P01806	similar to Ig heavy chain V region 441 precursor; immunoglobulin heavy chain (X24 family)

Supplementary Table S18. DE (down–regulated in L compared to U) genes in GO term cytoplasmic part.

ID	Gene Name
Q8BWU8	alanine-glyoxylate aminotransferase 2-like 1
Q64176	esterase 22
O88492	plasma membrane associated protein, S3-12
P12790	similar to testosterone 16a-hydroxylase type a; cytochrome P450, family 2, subfamily b, polypeptide 9

Supplementary Table S19. DE (down–regulated in H compared to U) genes in GO term category organic and metabolic processes.

ID	Gene Name
Q9QXE0	2-hydroxyacyl-CoA lyase 1
Q8JZV9	3-hydroxybutyrate dehydrogenase, type 2
O35490	Betaine--homocysteine S-methyltransferase 1
Q8VCH0	acetyl-Coenzyme A acyltransferase 1A
Q8VCH0	acetyl-Coenzyme A acyltransferase 1B
P41216	acyl-CoA synthetase long-chain family member 1
Q91WC3	acyl-CoA synthetase long-chain family member 6
Q9DBA8	amidohydrolase domain containing 1
Q91YI0	argininosuccinate lyase
P16460	argininosuccinate synthetase 1
Q8K4H1	arylformamidase
O35490	betaine-homocysteine methyltransferase
Q8C196	carbamoyl-phosphate synthetase 1
P23589	carbonic anhydrase 5a, mitochondrial
Q9JKJ9	cytochrome P450, family 39, subfamily a, polypeptide 1
Q9DBT9	dimethylglycine dehydrogenase precursor
Q9JLJ4	elongation of very long chain fatty acids (FEN1/Elo2, SUR4/Elo3, yeast)-like 2
O35949	elongation of very long chain fatty acids (FEN1/Elo2, SUR4/Elo3, yeast)-like 3
P34914	epoxide hydrolase 2, cytoplasmic
Q571F8	glutaminase 2 (liver, mitochondrial)
Q9QXF8	glycine N-methyltransferase
P50247	similar to Adenosylhomocysteinase (S-adenosyl-L-homocysteine hydrolase) (AdoHcyase) (Liver copper-binding protein) (CUBP); S-adenosylhomocysteine hydrolase
O88833	similar to DNA-directed RNA polymerase II 7.6 kDa polypeptide (RPB10) (RPB7.6) (RPABC5); hypothetical protein LOC100044218; predicted gene 13015; polymerase (RNA) II (DNA directed) polypeptide L; cytochrome P450, family 4, subfamily a, polypeptide 31; cytochrome P450, family 4, subfamily a, polypeptide 32; predicted gene 10774; cytochrome P450, family 4, subfamily a, polypeptide 10

Q62452	similar to UDP glycosyltransferase 1 family polypeptide A13; similar to UGT1.6; UDP glucuronosyltransferase 1 family, polypeptide A1; UDP glucuronosyltransferase 1 family, polypeptide A2; UDP glycosyltransferase 1 family, polypeptide A10; UDP glucuronosyltransferase 1 family, polypeptide A5; UDP glycosyltransferase 1 family, polypeptide A cluster; UDP glucuronosyltransferase 1 family, polypeptide A9; UDP glucuronosyltransferase 1 family, polypeptide A8; similar to UDP glycosyltransferase 1 family, polypeptide A8; UDP glucuronosyltransferase 1 family, polypeptide A7C; UDP glucuronosyltransferase 1 family, polypeptide A6A; similar to UDP glucuronosyltransferase 1 family, polypeptide A6B; UDP glucuronosyltransferase 1 family, polypeptide A6B
Q8BS35	transmembrane protein 195
Q8QZR1	tyrosine aminotransferase
Q8VC12	urocanase domain containing 1

Supplementary Table S20. KEGG pathway IDs over–expressed in high–infected livers compared to un–infected livers (U vs H).

KEGG ID: Description	P value (FDR corrected)
mmu04062:Chemokine signaling pathway	1.41E-08
mmu04110:Cell cycle	2.84E-08
mmu04060:Cytokine-cytokine receptor interaction	2.91E-08
mmu04640:Hematopoietic cell lineage	1.39E-07
mmu05340:Primary immunodeficiency	8.40E-07
mmu04514:Cell adhesion molecules (CAMs)	6.10E-06
mmu04670:Leukocyte transendothelial migration	1.19E-05
mmu04510:Focal adhesion	5.16E-05
mmu04650:Natural killer cell mediated cytotoxicity	5.31E-05
mmu04620:Toll-like receptor signaling pathway	1.65E-04
mmu04621:NOD-like receptor signaling pathway	1.75E-04
mmu03030:DNA replication	2.90E-04
mmu05332:Graft-versus-host disease	5.49E-04
mmu05330:Allograft rejection	0.001744
mmu04672:Intestinal immune network for IgA production	0.001775
mmu04660:T cell receptor signaling pathway	0.001824
mmu04115:p53 signaling pathway	0.002112
mmu04662:B cell receptor signaling pathway	0.002591
mmu04666:Fc gamma R-mediated phagocytosis	0.004076
mmu04512:ECM-receptor interaction	0.004189
mmu04914:Progesterone-mediated oocyte maturation	0.005762
mmu04144:Endocytosis	0.00603
mmu05320:Autoimmune thyroid disease	0.0081
mmu04940:Type I diabetes mellitus	0.009566
mmu05200:Pathways in cancer	0.009722
mmu05222:Small cell lung cancer	0.011396
mmu04612:Antigen processing and presentation	0.01223

mmu05414:Dilated cardiomyopathy	0.013755
mmu04210:Apoptosis	0.014071
mmu04010:MAPK signaling pathway	0.018224
mmu04810:Regulation of actin cytoskeleton	0.01905
mmu04722:Neurotrophin signaling pathway	0.021964
mmu00520:Amino sugar and nucleotide sugar metabolism	0.023069
mmu04360:Axon guidance	0.023214
mmu05220:Chronic myeloid leukemia	0.025735
mmu04630:Jak-STAT signaling pathway	0.03008
mmu05212:Pancreatic cancer	0.03014
mmu03010:Ribosome	0.030881
mmu05416:Viral myocarditis	0.030925
mmu00052:Galactose metabolism	0.031272
mmu05310:Asthma	0.041627

Supplementary Table S21. KEGG pathway IDs under–expressed in high–infected livers compared to un–infected livers (U vs H).

KEGG ID: Description	P-value (FDR corrected)
mmu00280:Valine, leucine and isoleucine degradation	4.33E-12
mmu00190:Oxidative phosphorylation	5.37E-12
mmu00071:Fatty acid metabolism	6.06E-12
mmu00982:Drug metabolism	4.40E-11
mmu05012:Parkinson's disease	4.58E-10
mmu03320:PPAR signaling pathway	5.31E-09
mmu00980:Metabolism of non-targets by cytochrome P450	1.80E-08
mmu00380:Tryptophan metabolism	1.40E-07
mmu00640:Propanoate metabolism	1.47E-07
mmu05010:Alzheimer's disease	2.74E-07
mmu05016:Huntington's disease	3.03E-07
mmu00650:Butanoate metabolism	6.32E-06
mmu00310:Lysine degradation	3.44E-05
mmu00830:Retinol metabolism	3.84E-05
mmu00010:Glycolysis / Gluconeogenesis	3.84E-05
mmu00983:Drug metabolism	8.37E-05
mmu00140:Steroid hormone biosynthesis	5.51E-04
mmu00350:Tyrosine metabolism	0.001226
mmu00410:beta-Alanine metabolism	0.001247
mmu00860:Porphyrin and chlorophyll metabolism	0.001257
mmu00120:Primary bile acid biosynthesis	0.001373
mmu00020:Citrate cycle (TCA cycle)	0.001477
mmu01040:Biosynthesis of unsaturated fatty acids	0.001548
mmu00500:Starch and sucrose metabolism	0.001648
mmu00260:Glycine, serine and threonine metabolism	0.00184
mmu00330:Arginine and proline metabolism	0.00313
mmu00030:Pentose phosphate pathway	0.004465

mmu00620:Pyruvate metabolism	0.00595
mmu00340:Histidine metabolism	0.01335
mmu04610:Complement and coagulation cascades	0.02252
mmu00051:Fructose and mannose metabolism	0.023237
mmu00130:Ubiquinone and other terpenoid-quinone biosynthesis	0.034972
mmu00903:Limonene and pinene degradation	0.036754
mmu00053:Ascorbate and aldarate metabolism	0.036754
mmu00591:Linoleic acid metabolism	0.043429
mmu00250:Alanine, aspartate and glutamate metabolism	0.044523
mmu00630:Glyoxylate and dicarboxylate metabolism	0.048057

Supplementary Table S22. KEGG pathway IDs over–expressed in low–infected livers compared to un–infected livers (U vs L).

KEGG ID: Description	P value (FDR corrected)
mmu04062:Chemokine signaling pathway	1.59E-09
mmu04514:Cell adhesion molecules (CAMs)	5.40E-07
mmu04060:Cytokine-cytokine receptor interaction	3.90E-06
mmu04510:Focal adhesion	9.34E-06
mmu04620:Toll-like receptor signaling pathway	9.88E-06
mmu04650:Natural killer cell mediated cytotoxicity	1.37E-05
mmu04640:Hematopoietic cell lineage	1.87E-05
mmu05332:Graft-versus-host disease	5.57E-05
mmu04670:Leukocyte transendothelial migration	6.33E-05
mmu04512:ECM-receptor interaction	1.66E-04
mmu04612:Antigen processing and presentation	1.87E-04
mmu04621:NOD-like receptor signaling pathway	4.33E-04
mmu04110:Cell cycle	5.25E-04
mmu04210:Apoptosis	8.58E-04
mmu04666:Fc gamma R-mediated phagocytosis	0.00142
mmu05330:Allograft rejection	0.0022
mmu03030:DNA replication	0.003911
mmu04940:Type I diabetes mellitus	0.004629
mmu05340:Primary immunodeficiency	0.004739
mmu04662:B cell receptor signaling pathway	0.00627
mmu04672:Intestinal immune network for IgA production	0.01033
mmu04660:T cell receptor signaling pathway	0.011434
mmu04623:Cytosolic DNA-sensing pathway	0.011676
mmu05416:Viral myocarditis	0.011745
mmu04810:Regulation of actin cytoskeleton	0.012546
mmu05320:Autoimmune thyroid disease	0.013436
mmu04630:Jak-STAT signaling pathway	0.020912

mmu05222:Small cell lung cancer	0.02348
mmu00052:Galactose metabolism	0.030874

Supplementary Table S23. KEGG pathway IDs under–expressed in low–infected livers compared to un–infected livers (U vs L).

KEGG ID: Description	P value (FDR corrected)
mmu00280:Valine, leucine and isoleucine degradation	1.20E-17
mmu00982:Drug metabolism	1.62E-10
mmu00640:Propanoate metabolism	2.19E-10
mmu05012:Parkinson's disease	2.42E-10
mmu00190:Oxidative phosphorylation	5.19E-10
mmu00071:Fatty acid metabolism	2.25E-09
mmu05016:Huntington's disease	1.82E-08
mmu00650:Butanoate metabolism	4.72E-08
mmu00980:Metabolism of non-targets by cytochrome P450	4.80E-08
mmu00310:Lysine degradation	2.27E-07
mmu05010:Alzheimer's disease	5.16E-07
mmu00380:Tryptophan metabolism	1.12E-06
mmu00410:beta-Alanine metabolism	4.41E-06
mmu03320:PPAR signaling pathway	9.03E-05
mmu00260:Glycine, serine and threonine metabolism	2.13E-04
mmu01040:Biosynthesis of unsaturated fatty acids	2.94E-04
mmu00983:Drug metabolism	3.43E-04
mmu00330:Arginine and proline metabolism	9.26E-04
mmu00340:Histidine metabolism	9.70E-04
mmu00830:Retinol metabolism	0.002648
mmu00250:Alanine, aspartate and glutamate metabolism	0.003571
mmu00020:Citrate cycle (TCA cycle)	0.00434
mmu00620:Pyruvate metabolism	0.006816
mmu00500:Starch and sucrose metabolism	0.011462
mmu00053:Ascorbate and aldarate metabolism	0.012395
mmu00770:Pantothenate and CoA biosynthesis	0.012395
mmu00903:Limonene and pinene degradation	0.012395

mmu00860:Porphyrin and chlorophyll metabolism	0.015247
mmu00910:Nitrogen metabolism	0.015267
mmu00561:Glycerolipid metabolism	0.01541
mmu00350:Tyrosine metabolism	0.015716
mmu00630:Glyoxylate and dicarboxylate metabolism	0.015802
mmu00010:Glycolysis / Gluconeogenesis	0.019688
mmu00140:Steroid hormone biosynthesis	0.036536

Supplementary Table S24. KEGG pathway IDs over–expressed in high–infected livers compared to low–infected livers (L vs H).

KEGG ID: Description	P-value (FDR corrected)
mmu04512:ECM-receptor interaction	1.46E-05
mmu03010:Ribosome	1.70E-05
mmu04110:Cell cycle	1.42E-04
mmu04510:Focal adhesion	0.001061
mmu04060:Cytokine-cytokine receptor interaction	0.028856

Supplementary Table S25. KEGG pathway IDs under–expressed in high–infected livers compared to low–infected livers (L vs H).

KEGG ID: Description	P-value (FDR corrected)
mmu00982:Drug metabolism	2.67E-11
mmu00980:Metabolism of non-targets by cytochrome P450	1.16E-10
mmu00280:Valine, leucine and isoleucine degradation	2.98E-04
mmu04910:Insulin signaling pathway	5.43E-04
mmu00500:Starch and sucrose metabolism	0.001326
mmu00830:Retinol metabolism	0.004386
mmu00140:Steroid hormone biosynthesis	0.004395
mmu00591:Linoleic acid metabolism	0.004578
mmu00150:Androgen and estrogen metabolism	0.004588
mmu00350:Tyrosine metabolism	0.008158
mmu00640:Propanoate metabolism	0.008634
mmu00250:Alanine, aspartate and glutamate metabolism	0.008634
mmu00380:Tryptophan metabolism	0.008703
mmu00620:Pyruvate metabolism	0.009314
mmu00010:Glycolysis / Gluconeogenesis	0.010026
mmu00983:Drug metabolism	0.01941
mmu00650:Butanoate metabolism	0.021895
mmu00480:Glutathione metabolism	0.025795
mmu01040:Biosynthesis of unsaturated fatty acids	0.026139
mmu00260:Glycine, serine and threonine metabolism	0.049532

Supplementary Table S26. Fold change (Log_2 fold change) of immune response related genes identified from in significantly over expressed genes sets.

UH: U vs H; UL: U vs L; LH: L vs H

#: Components of Th1 immune response

*: Components of Th1 immune response

Cells are color coded based on fold change.

High fold change Low fold change

Immune system component (cytokines)	Up–regulated		
	**UH**	**UL**	**LH**
#TNF–α: Cause severe hepatic fibrosis	15.3576	6.10146	
#IFN–γ: Negatively regulates Th2 response and fibrosis	112.629	91.7533	0.825
#IL1: Down regulation of granulomatous response	10.9194	8.75972	
*IL4: Promotes Th2 response and cell recruitment	6.21665	4.24213	
#IL6: Th1 immune response	1.3319		
*IL10: Regulates Th1/Th2 balance	5.63199	1.66903	
IL2	12.209	4.94659	
IL3	5.83515	4.09702	
IL7	2.73733		
IL12	3.49419		
IL16	2.20902	1.4652	
IL18	4.26782	4.27638	
IL21	3.81339		
IL27	2.78834	1.78176	
IL31	2.40349		
IL33	3.44777		1.21332
IG–E	3.443167		2.744416

Supplementary Figures

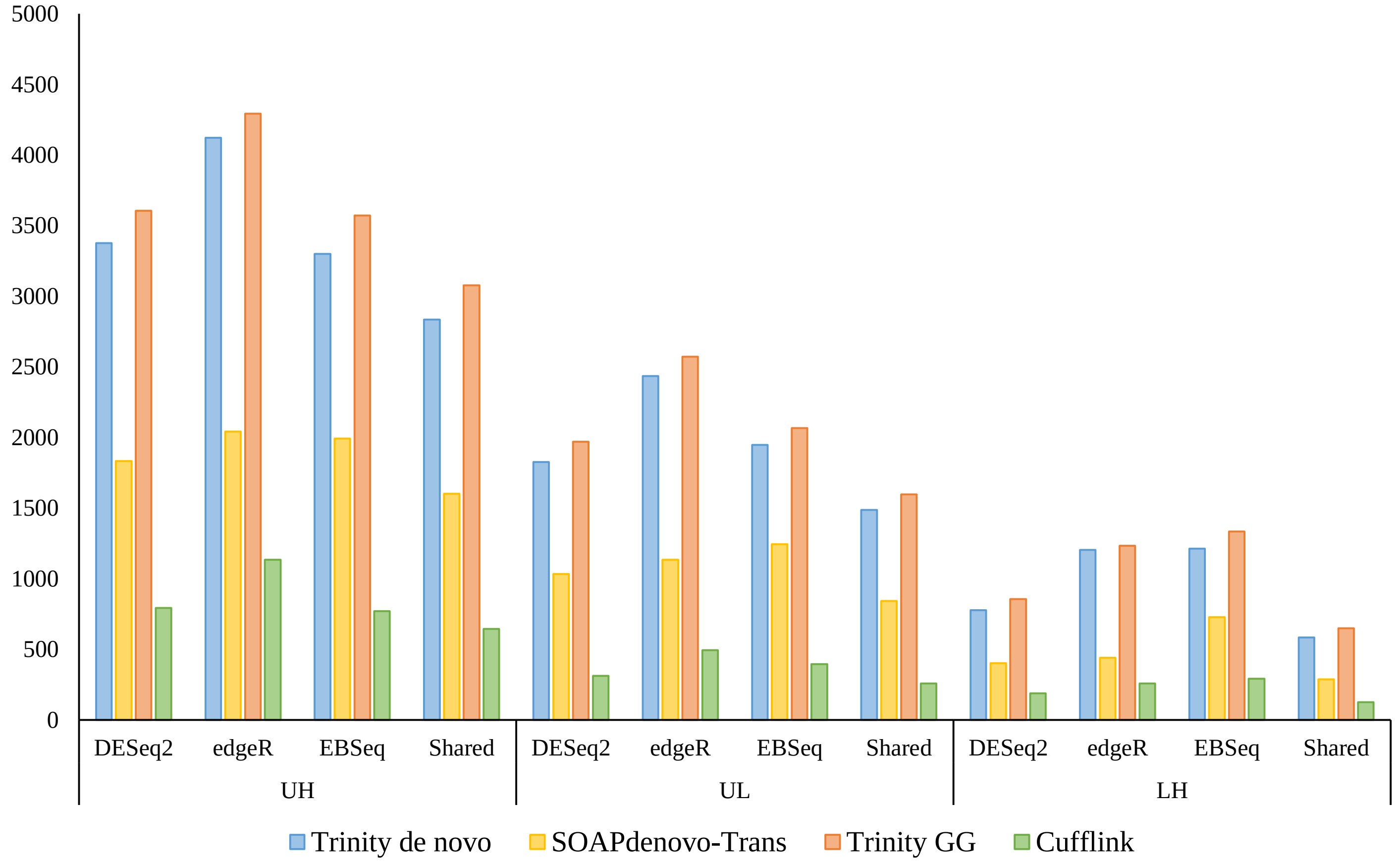

Supplementary Figure S1. The number of DE genes predicted by DESeq2, edgeR and EBSeq. These include differentially predicted non-target hits. Reference guided Trinity assemblies produced the most DE genes after Benjamini–Hochberg correction. As expected, programs DESeq2 and edgeRE were more conservative in identifying DE genes and EBSeq predicted the most. DESeq2 and EBSeq shared the most DE predictions
UH: H vs U; UL: L vs U; LH: H vs L
Shared: Genes predicted as DE by all three DE analysis software

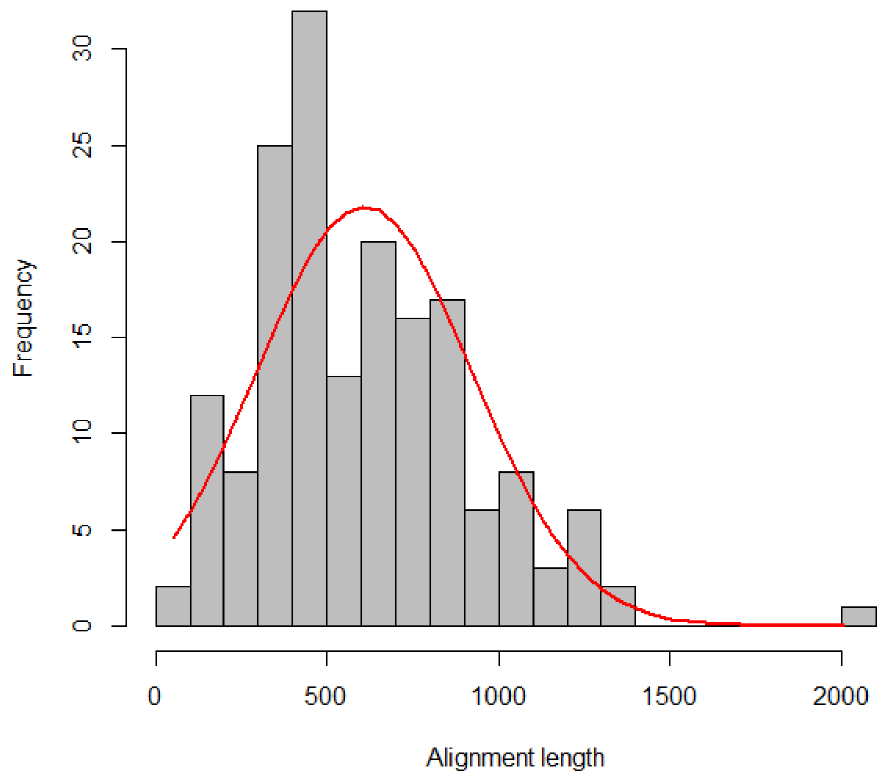

Supplementary Figure S2. Alignment length distribution of differentially expressed non-target reads. Source: Trinity *de novo* and SOAPdenovo-Trans assemblies (Figure 2).

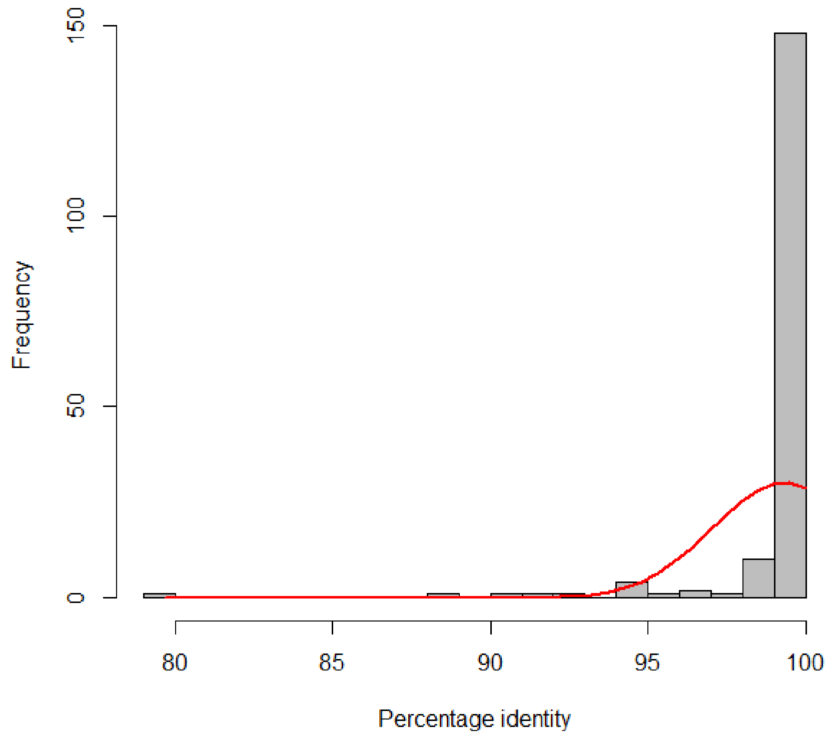

Supplementary Figure S3. Percentage identity distribution of differentially expressed non-target reads. Source: Trinity *de novo* and SOAPdenovo-Trans assemblies (Figure 2).

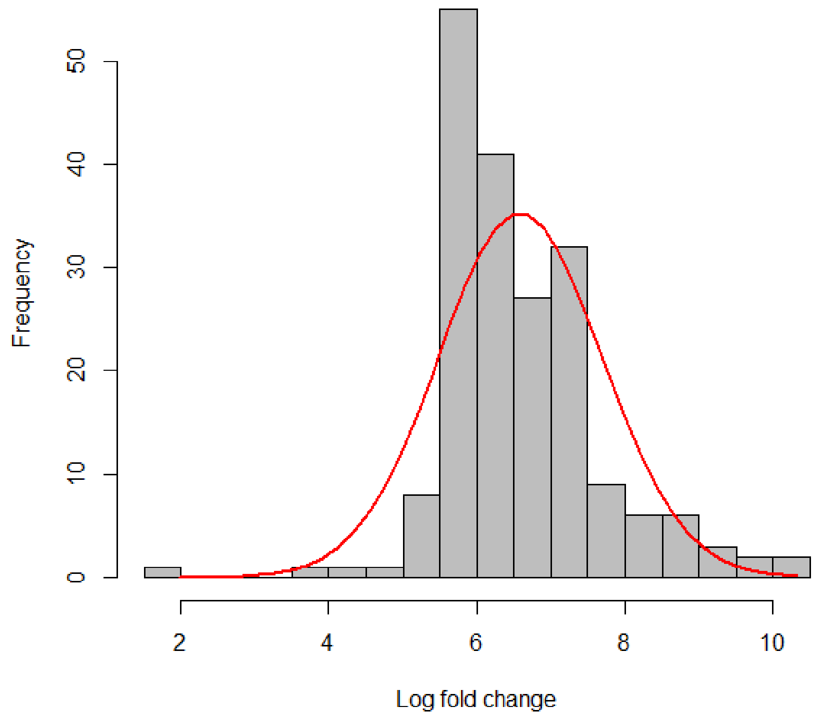

Supplementary Figure S4. Fold change (Log_2 fold change) distribution of differentially expressed non-target reads. Source: Trinity *de novo* and SOAPdenovo-Trans assemblies (Figure 2).

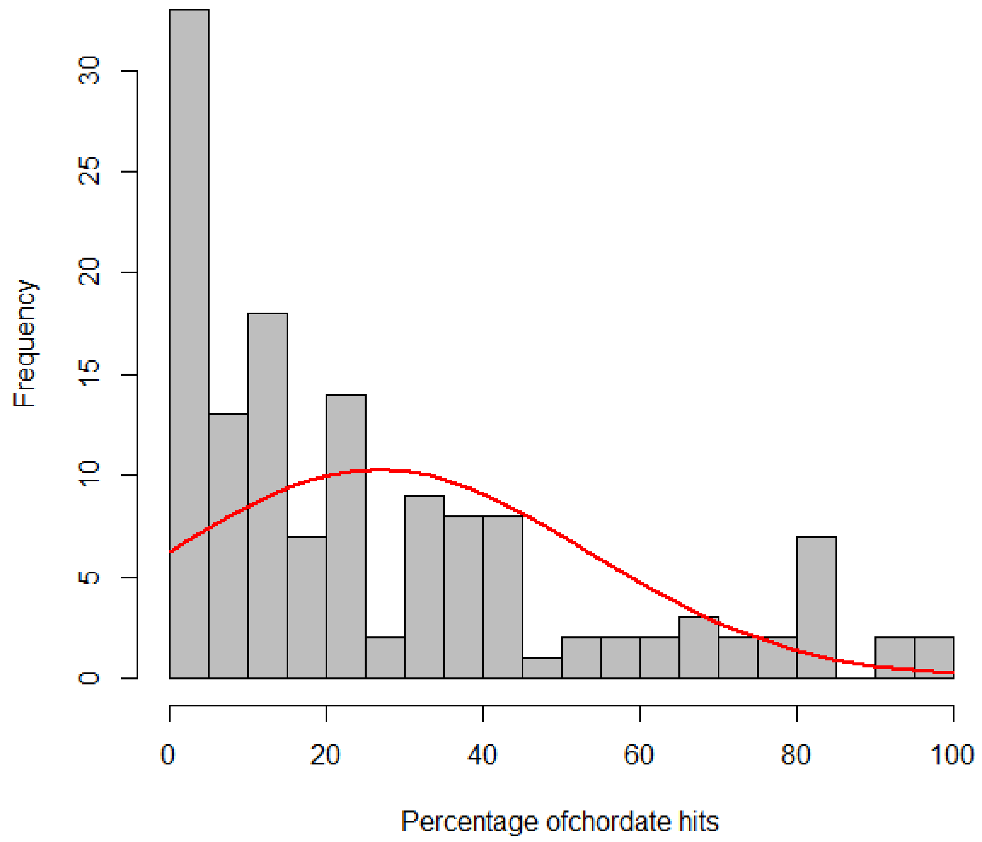

Supplementary Figure S5. Percentage of chordate hits significantly matched with each differentially expressed non-target sequence (BLASTX against swissprot database; E–value 10–6; ≥90% percentage ID; 1000 hits per sequence). Source: Trinity *de novo* and SOAPdenovo-Trans assemblies (Figure 2).

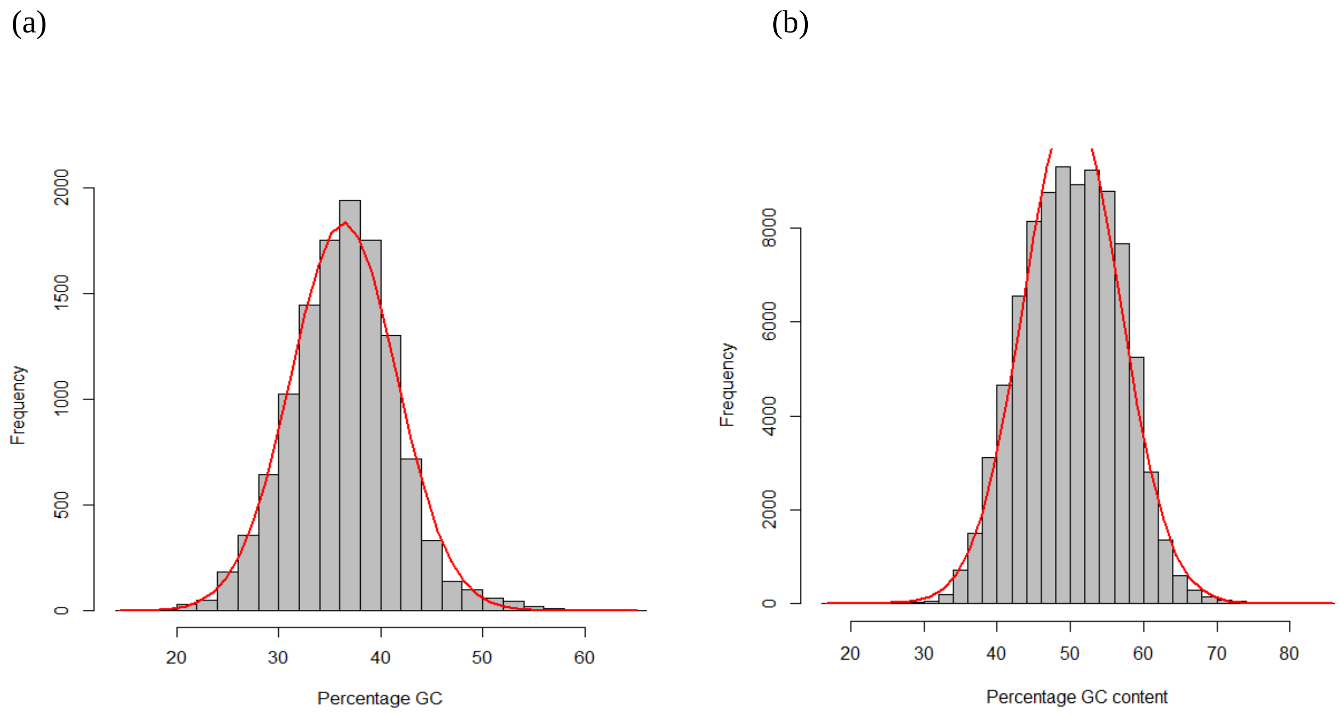
(a)
Frequency
0
500
1000
1500
2000
20
30
40
50
60
Percentage GC
(b)
Frequency
0
2000
4000
6000
8000
20
30
40
50
60
70
80
Percentage GC content

(c)

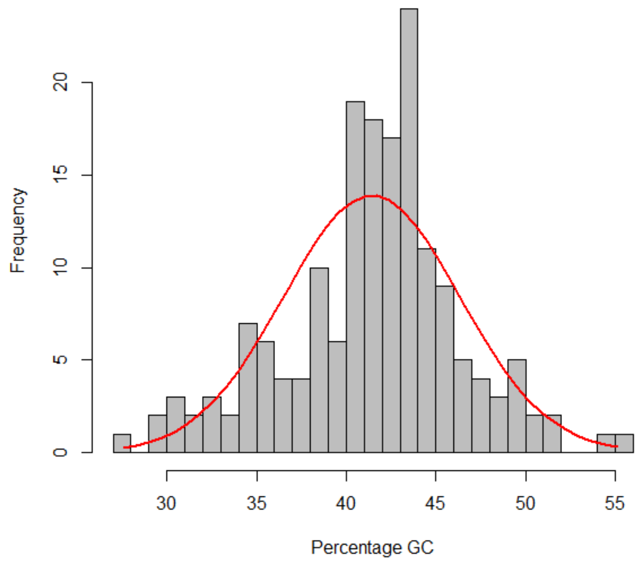

Supplementary Figure S6. Percentage GC content of (a) *S. mansoni* cDNA (Average 36.4) (b) *Mus musculus* cDNA (Average 50.2) (c) Differentially expressed non-target sequences of *S. mansoni* origin (Average 41.4). Source: Trinity *de novo* and SOAPdenovo-Trans assemblies (Figure 2).

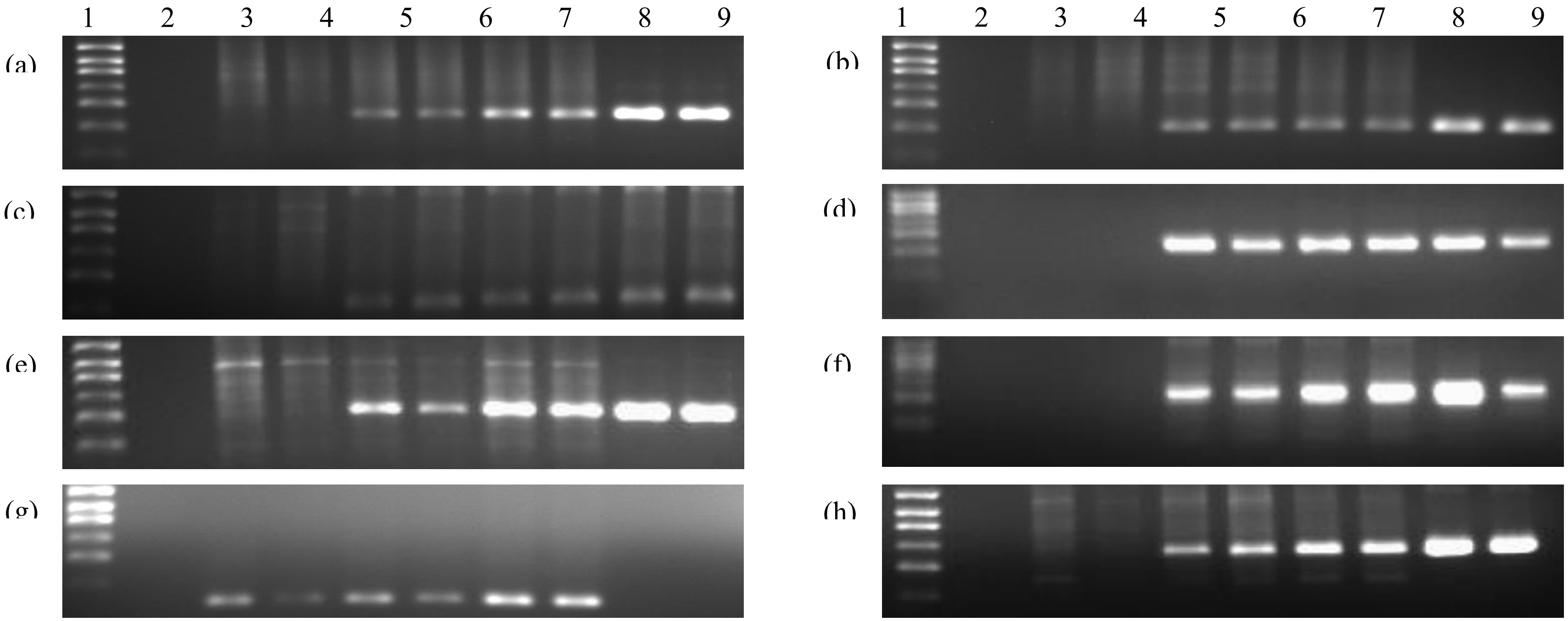

Supplementary Figure S7. PCR amplification of non-target reads. Ethidium bromide stained 1.5% agarose gels with bands corresponding to PCR products, (a) Contig 437 (b) Contig c92104 (c) Contig c93270 (d) Scaffold 152316 (e) Contig 296945 (f) Contig 18347 (g) vertebrate specific sequence (Kelly et al., 2014) (h) *S. mansoni* specific sequence (Grunau and Boissier, 2010). Lanes 1: agarose ladder (size bands: 738 bp, 597 bp, 508 bp, 401 bp, 305 bp, 200 bp, 104 bp), 2: negative control, 3: mouse cDNA U1, 4: mouse cDNA U2*,* 5*:* mouse cDNA L1*,* 6: mouse cDNA L2. 7: mouse cDNA H1*,* 8: mouse cDNA H2*,* 9: *S. mansoni* cDNA sample 1*,* 10: *S. mansoni* cDNA sample 2.

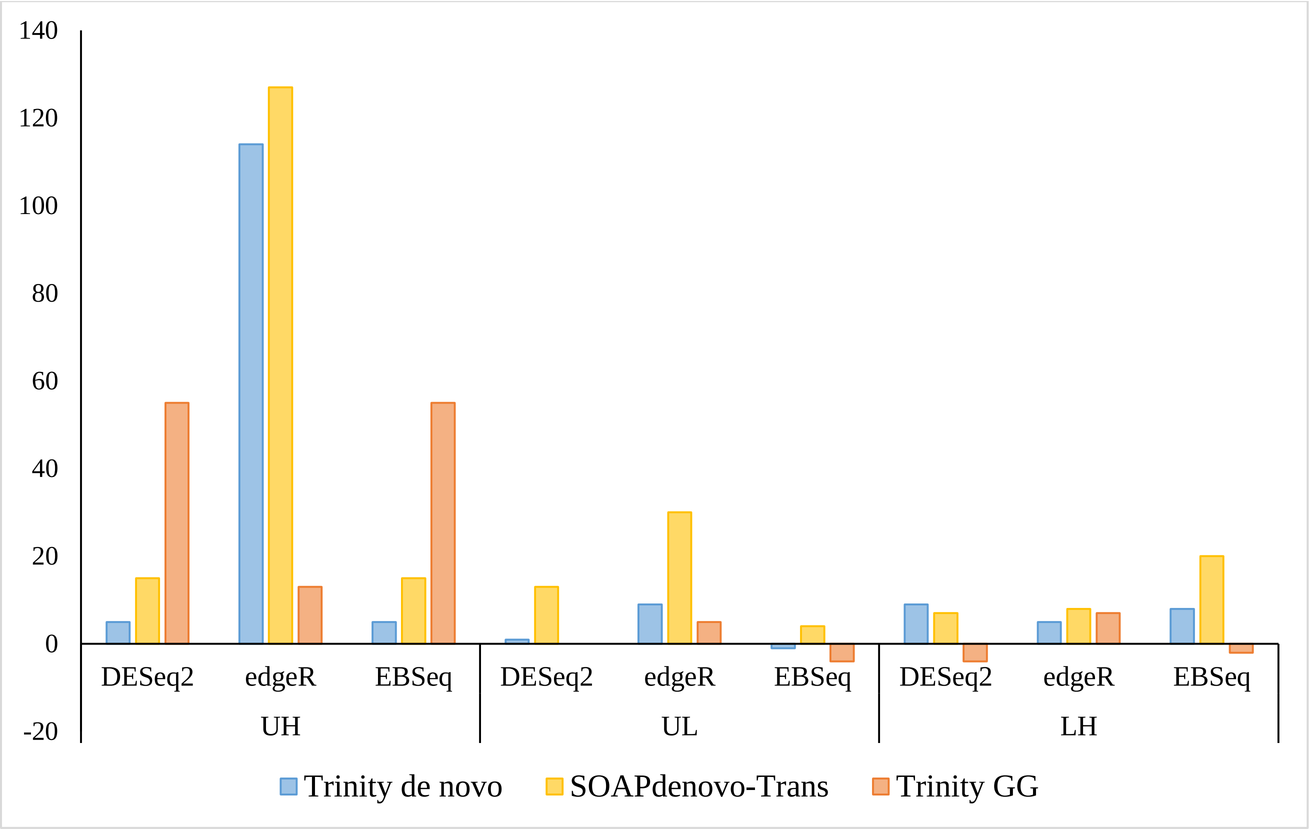

Supplementary Figure S8. The Change in number of DE transcripts after removing the reads of non-target origin. X–axis: DE genes before filtering non-targets – DE genes after removing non-targets

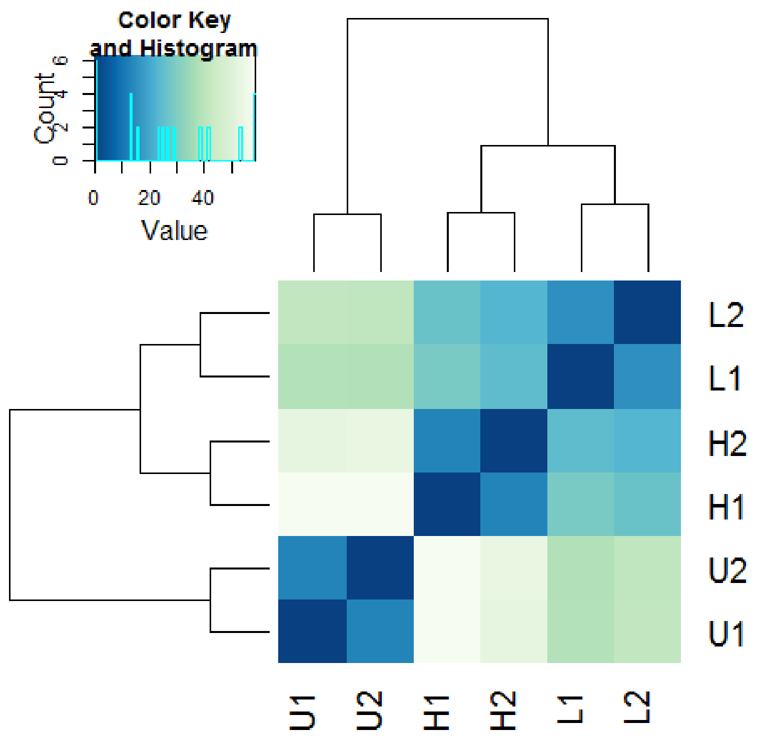

Supplementary Figure S9. Heat map representing sample to sample Euclidian distance calculated from gene–count matrix. Count data were log–transformed before distance calculation, using DESeq2, Trinity *de novo* assembly
U1, U2: Uninfected liver; L1, L2: Low–infected liver; H1, H2: High–infected liver

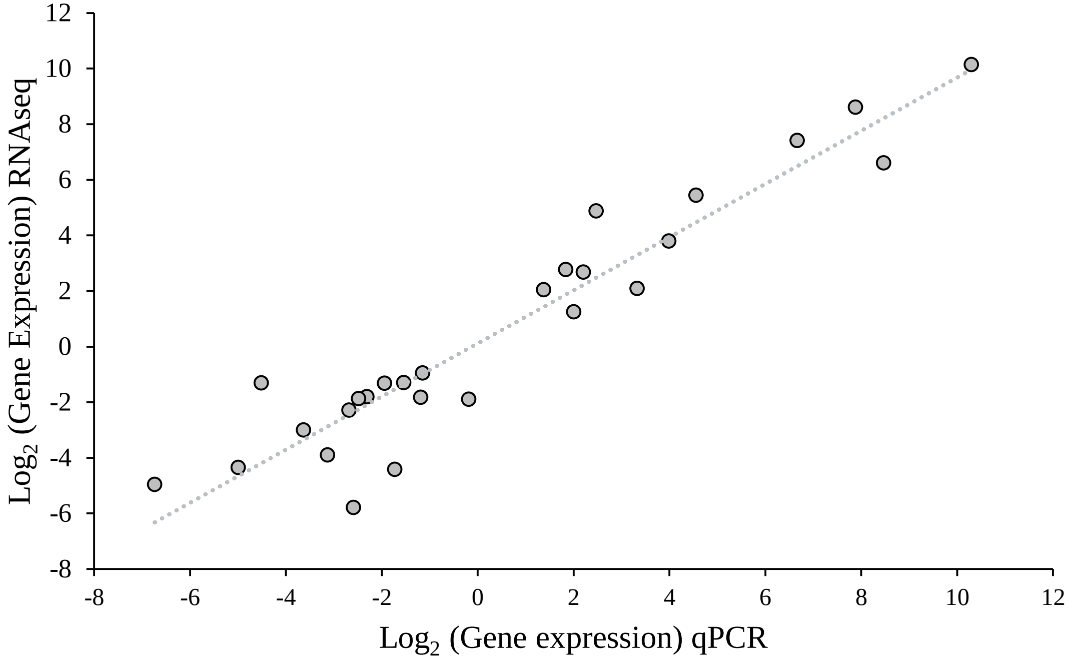

Supplementary Figure S10. Correlation of RNAseq (Trnity *de novo* assembly, DESeq2 ratios) reads and qPCR derived gene expression ratios. Pearson correlation coefficient: 0.9

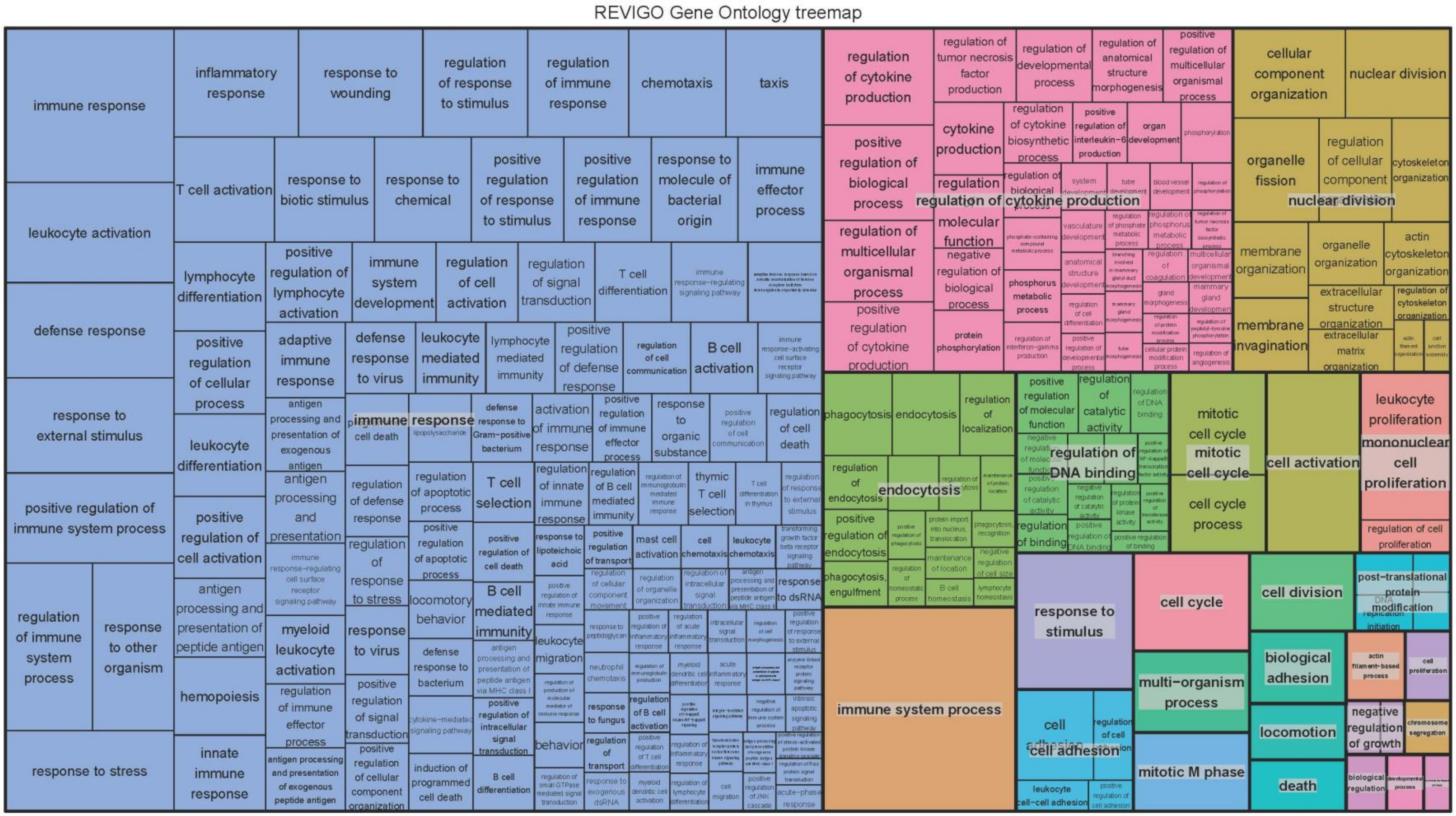

Supplementary Figure S11. GO “Biological processes” significantly over–expressed in low– infected livers compared to the uninfected livers (U vs. H). Each rectangle represents a GO category cluster. All the rectangles of one color belong to a single super cluster. The size of the square represents the log p value of the each GO cluster. The figure adapted from REVIGO software (Supek et al, 2011).

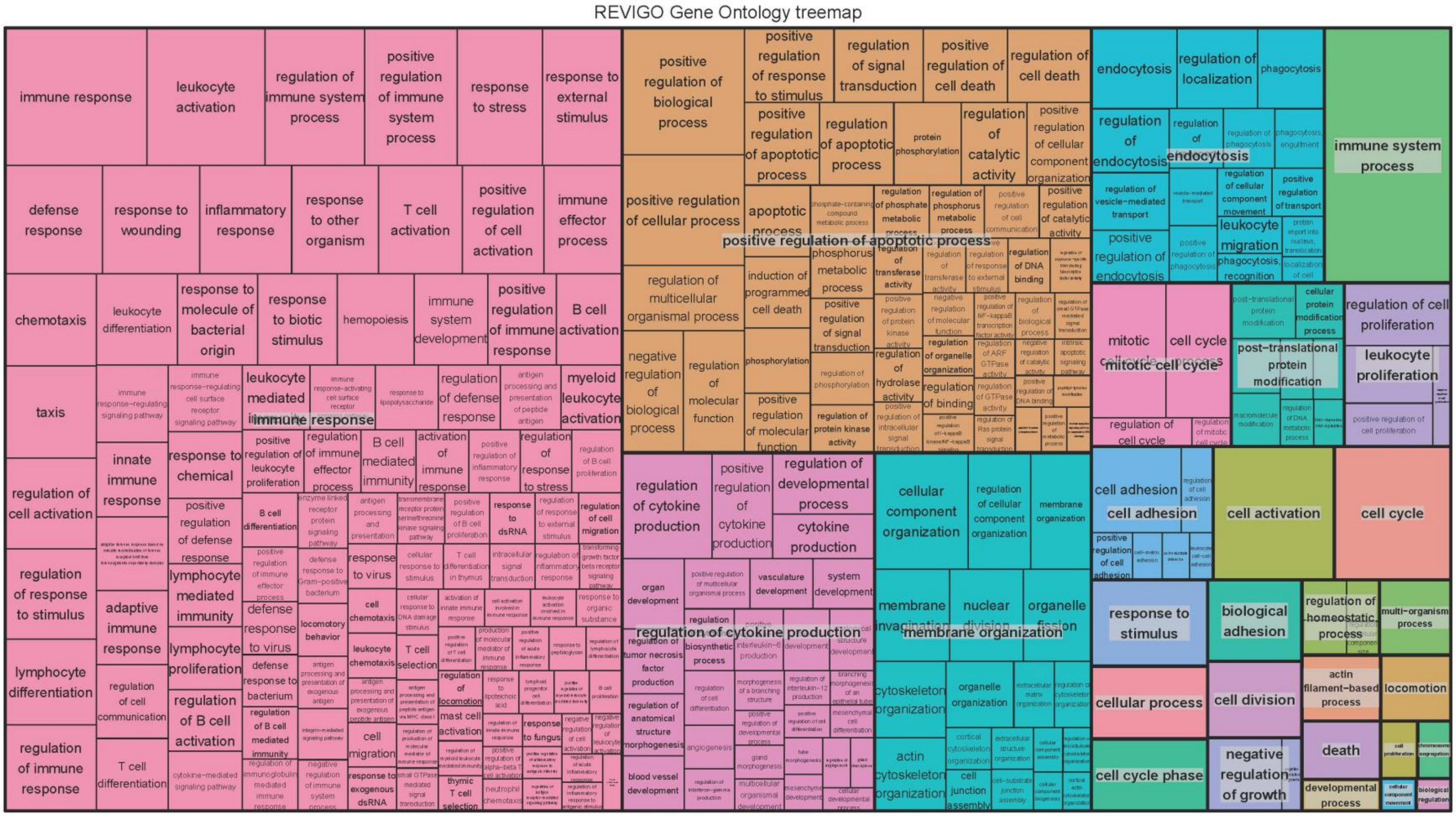

Supplementary Figure S12. GO "Biological processes" significantly over–expressed in highly infected livers compared to the uninfected livers (U vs. L).

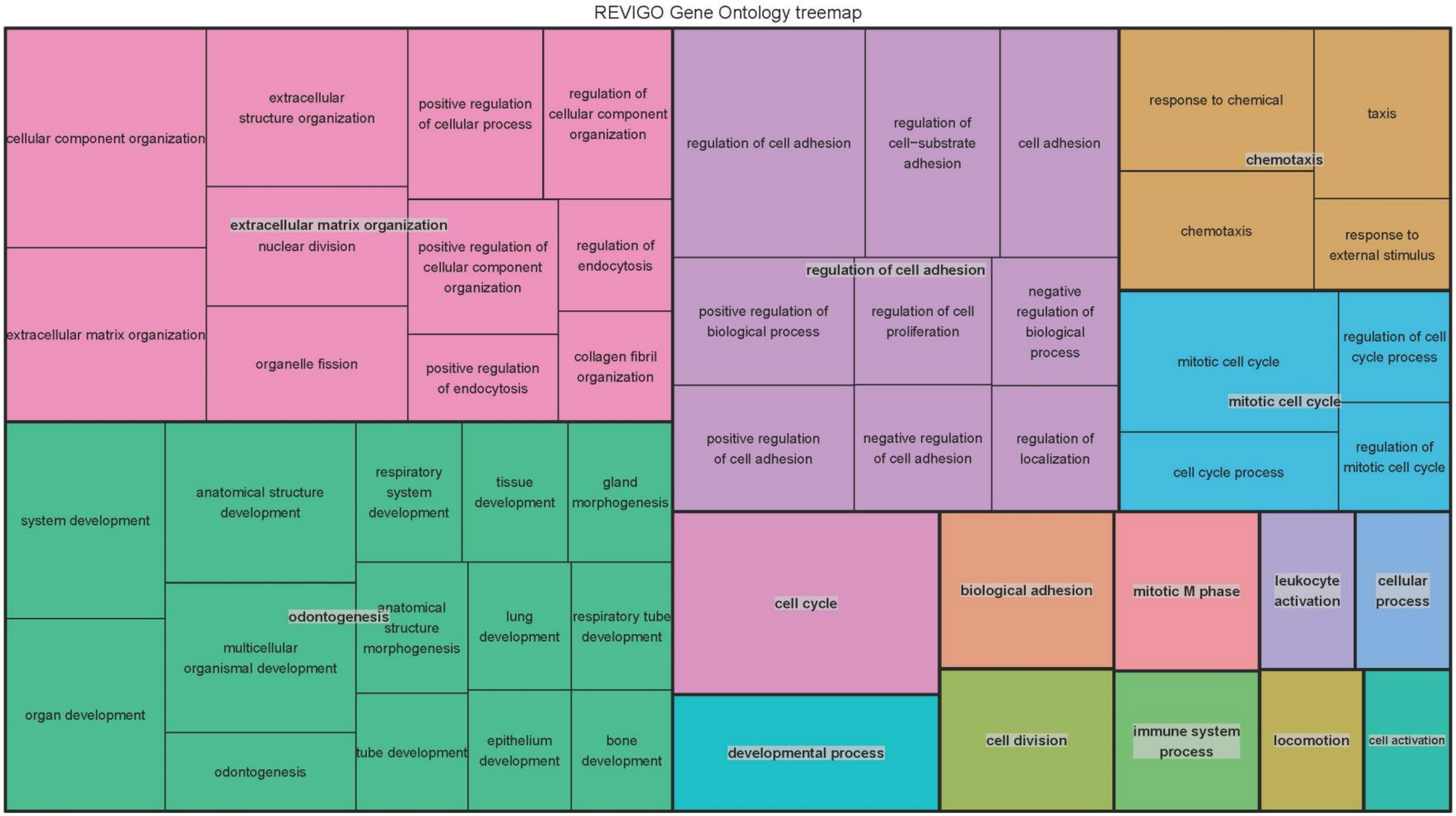

Supplementary Figure S13. GO "Biological processes" significantly over–expressed in high–infected livers compared to the low–infected livers (L vs. H).

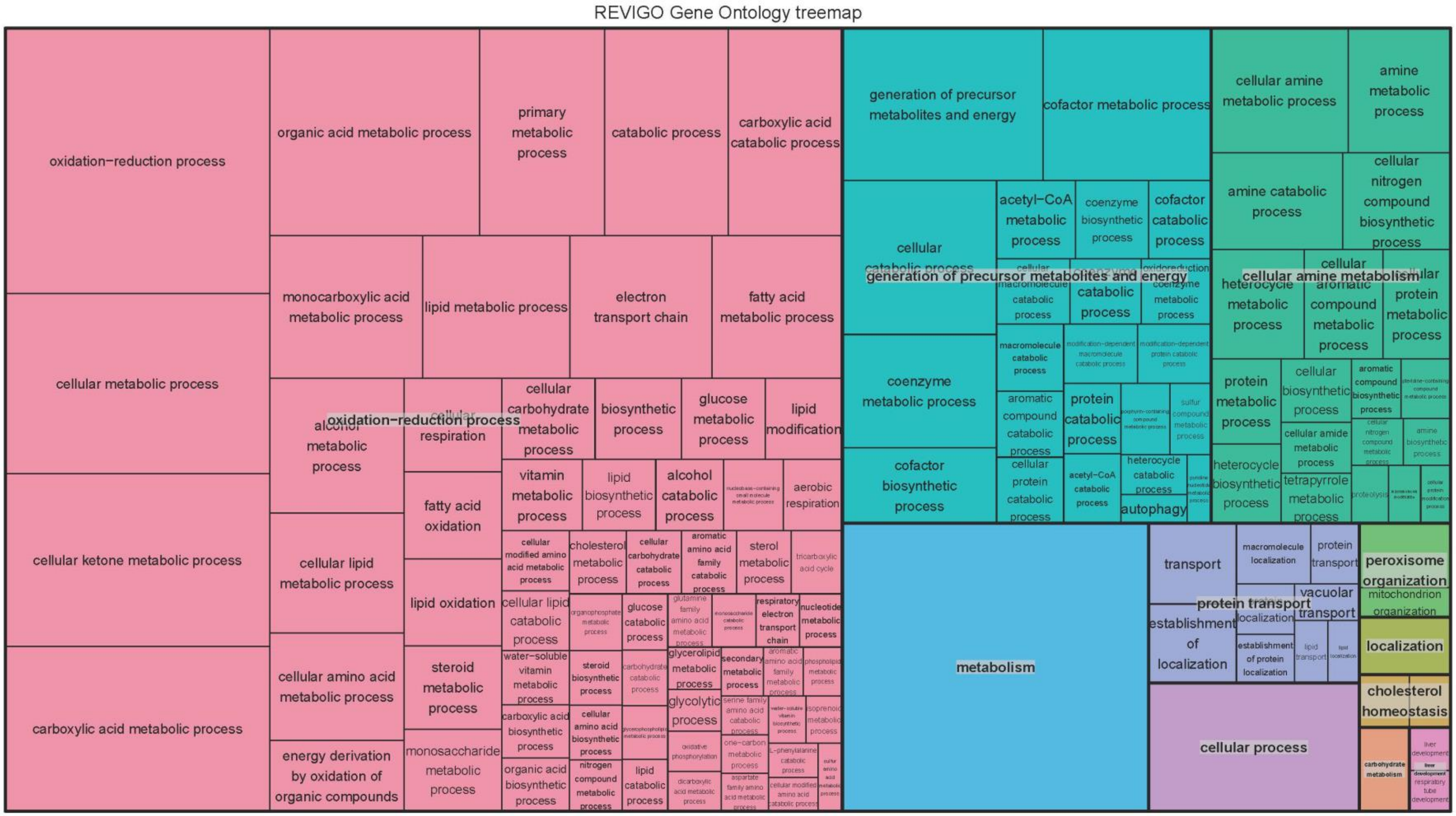

Supplementary Figure S14. GO "Biological processes" significantly under–expressed in highly infected livers compared to the uninfected livers (U vs. H).

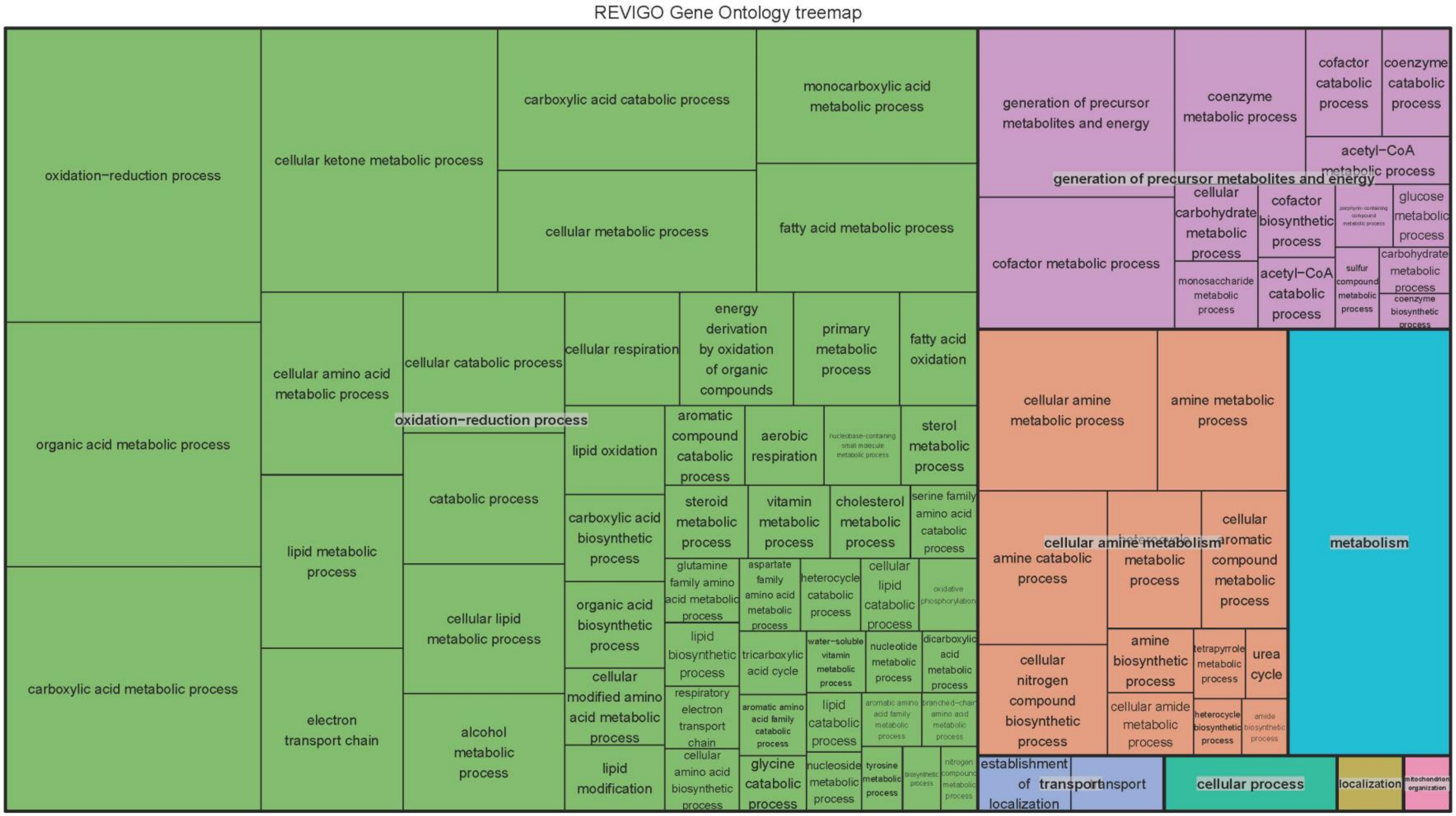

Supplementary Figure S15. GO "Biological processes" significantly under–expressed in low– infected livers compared to the uninfected livers (U vs. L).

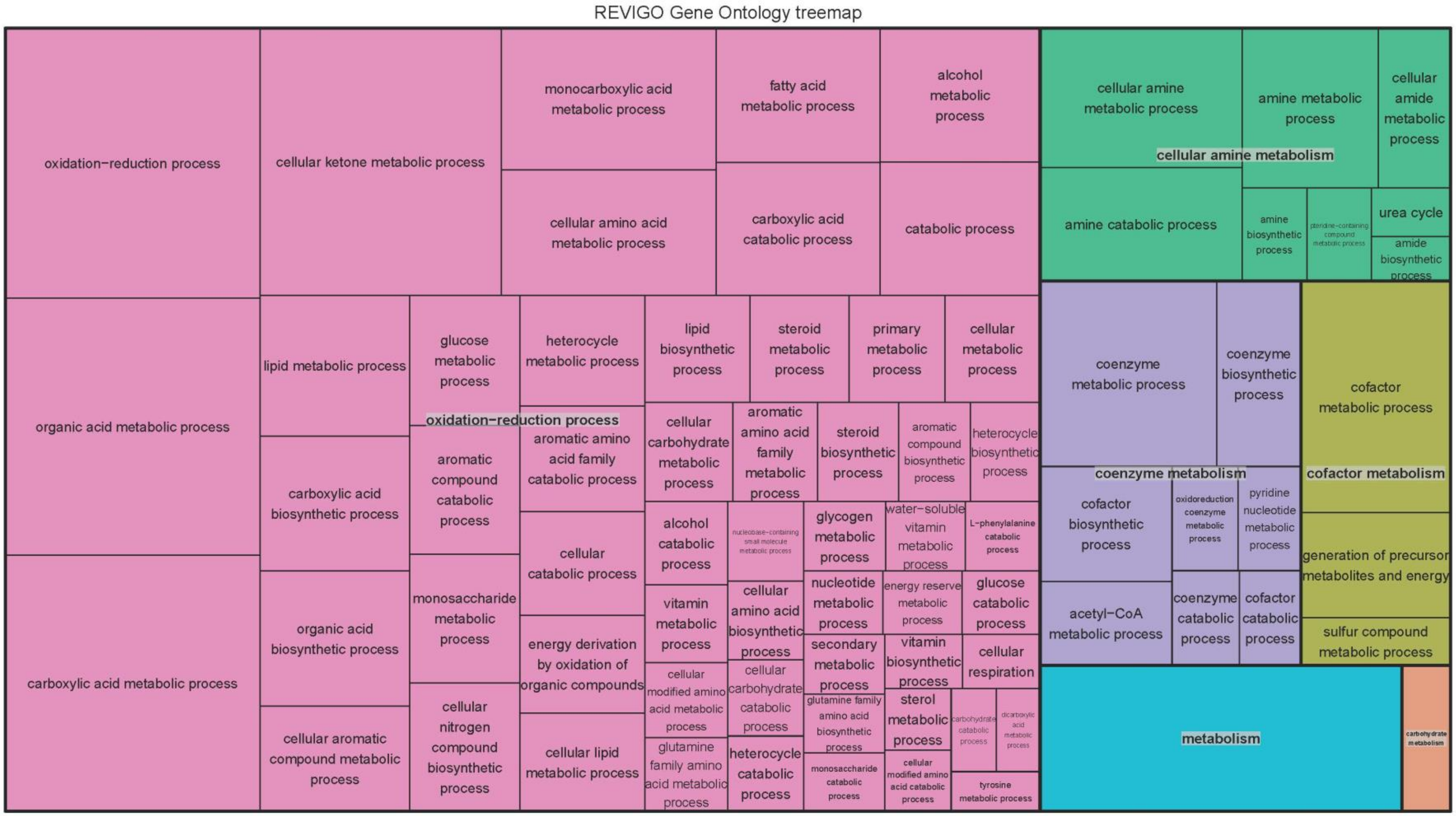

Supplementary Figure S16. GO "Biological processes" significantly under–expressed in highly infected livers compared to the low–infected livers (L vs. H).
